# Using public participation to sample trace metals in lake surface sediments: the OPAL Metals Survey

**DOI:** 10.1007/s10661-017-5946-y

**Published:** 2017-04-28

**Authors:** S. D. Turner, N. L. Rose, B. Goldsmith, J. M. Bearcock, C. Scheib, H. Yang

**Affiliations:** 1 Environmental Change Research Centre, UCL Geography, Gower St, London, WC1E 6BT UK; 2 Environmental Science Centre, British Geological Survey, Nicker Hill, Keyworth, Nottingham, NG12 5GG UK

**Keywords:** Metals, Lakes, Citizen science, Sediment, Contamination

## Abstract

Members of the public in England were invited in 2010 to take part in a national metals survey, by collecting samples of littoral sediment from a standing water body for geochemical analysis. To our knowledge, this is the first national sediment metals survey using public participation and reveals a snapshot of the extent of metals contamination in ponds and lakes across England. Hg, Ni, Cu, Zn and Pb concentrations exceeding sediment quality guidelines for the health of aquatic biota are ubiquitous in ponds and lakes, not just in areas with a legacy of industrial activity. To validate the public sampling approach, a calibration exercise was conducted at ten water bodies selected to represent a range of lakes found across England. Sediment concentrations of Hg, Ni, Cu, Zn and Pb were measured in samples of soil, stream and littoral and deep water sediment to assess inputs. Significant differences between littoral sediment metal concentrations occur due to local variability, but also organic content, especially in upland, peat soil catchments. Variability of metal concentrations between littoral samples is shown to be low in small (<20 ha) lowland lakes. Larger and upland lakes with more complex inputs and variation in organic content of littoral samples have a greater variability. Collection of littoral sediments in small lakes and ponds, with or without voluntary participation, can provide a reliable sampling technique for the preliminary assessment of metal contamination in standing waters. However, the heterogeneity of geology, soils and history/extent of metal contamination in the English landscape, combined with the random nature of sample collection, shows that systematic sampling for evaluating the full extent of metal contamination in lakes is still required.

**Electronic supplementary material:**

The online version of this article (doi:10.1007/s10661-017-5946-y) contains supplementary material, which is available to authorized users.

## Introduction

Systematic, national-scale assessments of metal concentrations and potential risk of contamination in lakes and ponds are rare, not least due to the scale and logistics required in collecting representative samples. National/state-scale assessment of metals in lake sediments has occurred in Scandinavia and North America to assess deposition of long-range atmospheric fossil fuel emissions and wastewater inputs (Johansson 1989; Rognerud and Fjeld 1993; Verta et al. 1989; Wright and Henriksen 1978; Blocksom et al. 2002; Heiskary 1996; Kamman et al. 2004) and for geochemical reconnaissance for mineral exploration, e.g. in Canada (Friske and Hornbrook 1991; Painter et al. 1994). Soils and river sediment trace metal data in the UK have been assessed by the extensive and precise sampling completed by the British Geological Survey’s (BGS) Geochemical Baseline Survey of the Environment (G-BASE) project (Johnson and Breward 2004), but lake and pond metal concentrations have never been systematically surveyed at a national scale.

Co-operation, participation and communication between scientists and society are an indispensable component in modern environmental science. Public participation in environmental monitoring research has increased in recent years driven by both directed and grass root programmes of ‘environmental democratisation’ (Carolan 2006; Conrad and Hilchey 2011). The positive aspects of public participation within scientific programmes are undoubtedly many; however, there are limitations to what they may realistically achieve and these need to be considered and discussed openly (Riesch et al. 2013; Rose et al. 2016).

Public participation in aquatic science is not a modern phenomenon and has been used for aquatic monitoring and education programmes globally. In the UK, there is a history of water quality surveys with public involvement (Rose et al. 2016) and also more specific ecological and community directed monitoring programmes, e.g. The Riverfly Partnership (http://www.riverflies.org). In the USA and Canada, a broadly similar pattern can be seen with many volunteer networks undertaking national state and local assessments of water quality with variable scales of scientific complexity, participatory inclusiveness and length of monitoring periods (Canfield et al. 2002; Savan et al. 2003; Stokes et al. 1990).

Voluntary aquatic monitoring programmes overwhelmingly collect data by recording simple observations. By doing so, data collection needs to be engaging to broaden participation and require little training (e.g. Secchi depth measurements (http://www.secchidipin.org/), family level invertebrate identification (http://www.opalexplorenature.org/)) but is still able to provide reliable information relevant to the aims and objectives of the monitoring (Rose et al. 2016). These demands are not mutually exclusive; increased training improves data quality but requires investment in resources, while more detailed and complex data gathering reduces participation, due either to time required or educational or linguistic ability.

Due to their often complex and interactive nature, chemical and physical measurements are often consigned to variable quality ‘dip strip’ measures for pH, nitrates and phosphates, with limited quality control (Renberg and Hansson 2008; Au et al. 2000; Rose et al. 2016). However, with training and more involvement, volunteers and non-professional groups can collect and generate reliable environmental data (Au et al. 2000; Canfield et al. 2002).

Open Air Laboratories (OPAL) is a UK programme, launched in 2007 to bring scientists and communities together to deliver a research programme focused on three environmental themes: loss of biodiversity, environmental degradation and climate change. Through regional and national projects, people of all ages, abilities and backgrounds contribute to OPAL by observing and recording the world around them and submitting their data to national databases for analysis and interpretation (Davies et al. 2011). A series of surveys were produced to raise public awareness of science and nature and to encourage people to explore their local environment. The OPAL Water Survey was launched in 2010 to gain a snapshot assessment of water quality in England. This was achieved by using the presence/absence of easily identifiable classes of aquatic invertebrates and simple measures of water clarity and pH (Rose et al. 2016). As an addition to the main water survey, we used this opportunity of public engagement to generate a national-scale assessment of trace metal sediment concentrations in standing water bodies (lakes and ponds) termed the OPAL Metals Survey. To our knowledge, there have been no previous public participation projects involving trace metal assessment of freshwater sediments.

Metal concentrations in lake and pond sediments reflect the geology and chemistry of their catchments. Elevated levels of metals in sediments can occur from industrial and domestic sources that enter the lake from rivers and streams or directly from the atmosphere. Lakes and ponds store metals and other contaminants by being incorporated into sediments during deposition. Between entering a lake and long-term burial, metals may be incorporated into aquatic plants and organisms. Due to direct inputs or via trophic accumulation, elevated metal concentrations above that can be tolerated but have detrimental effect on growth and reproduction of organisms, causing or contributing to a decline in aquatic ecosystem health. Background or baseline values are essential to determine levels of contamination that affect freshwater systems. Analyses for these metals are not simple, making it problematic to involve untrained people in sampling and laboratory procedures, but the range of sites visited by volunteers is potentially more variable and numerous, without the significant investment required for expansive fieldwork and systematic geochemical sampling. Whereas standard lake sediment assessment for metals would usually use deep water cores (Rippey et al. 2008; Rose et al. 2012) and participants could not be expected to use a boat and coring equipment, so samples were requested from littoral areas around a lake or pond where safe access allowed.

The aim of this paper is twofold: first to assess the viability of using single littoral sediment samples for an assessment of whole lake metal contamination and, secondly, evaluate the results of this approach with publically submitted samples in the OPAL Metals Survey.

## Methods

The OPAL Metals Survey was promoted as an additional feature to the OPAL Water Survey whereby interested participants could obtain a survey pack by contacting the OPAL Water Centre at University College London (UCL). This approach was intended to improve sample returns and create a channel for participants to communicate issues, e.g. ask questions about sampling protocols, request extra packs, etc. Participants received an illustrated instruction sheet, a sheet for recording site details (site name, location, date and space for other information pertaining to the site and/or sample, i.e. possible contamination sources). The OPAL Water Survey provided guidance on how to find a lake and record the location, with suggestions of using a hand-held GPS but also by postcode or using online mapping. The decision of adding extra detail on the site (vegetation, evidence of disturbance, presence of pollution) was left to the individual sampler. A sealable bag for the sample, a pair of disposable gloves, a uniquely numbered label for the sample and a pre-addressed ‘Freepost’ ‘pillow’ envelope for return of the sample to UCL were also included in the package delivered to volunteers. Participants were asked to take a scoop of sediment from a safe and accessible littoral area using a suitable device (one suggestion involved using a cleaned stainless steel ladle on a stick), place the sample in the bag and to post it along with the datasheet to UCL. Participants were asked to obtain a sample of mud from the lake margin; being asked to look for ‘dark brown’ sediment and to ensure it was from ‘underwater’. Dark brown was mentioned to reduce sampling of very sandy/gravel sediments and ‘underwater’ to reduce collection of terrestrial/bankside soils. Safety guidelines with the pack also highlighted the need to collect samples from a safe and easily accessible position. Participants were not asked to investigate the whole lake and then collect what they thought to be the most representative, as we wanted to minimise effort for sample collection and hence increase return rates of samples. To encourage participant-led investigation in their local area, no limit was placed on the number of samples an individual could collect and no sampling strategy was suggested other than the instructions described above.

### Calibration sites and sampling methodology

Multi-element geochemical datasets are typically spatially dependent, with a range of different processes influencing the element abundances measured at each sample site (McQueen 2006). Spatial heterogeneity is the norm for lake sediments, heterogeneity increasing with scale/number of processes affecting the lake, i.e. variations in water depth, morphology of lake basins, inflow composition and littoral vegetation extent (Wang et al. 2014; Kumke et al. 2005; Hassan et al. 2010; Baudo et al. 1989; Abraham 1998). By asking participants to collect a single sample for analysis however, we infer that one littoral sample can be representative of a water body. A calibration exercise was therefore undertaken to quantify the spatial variability in littoral metal abundances and their relationship to soil, stream inflow and deep water sediments.

**Fig. 1:**
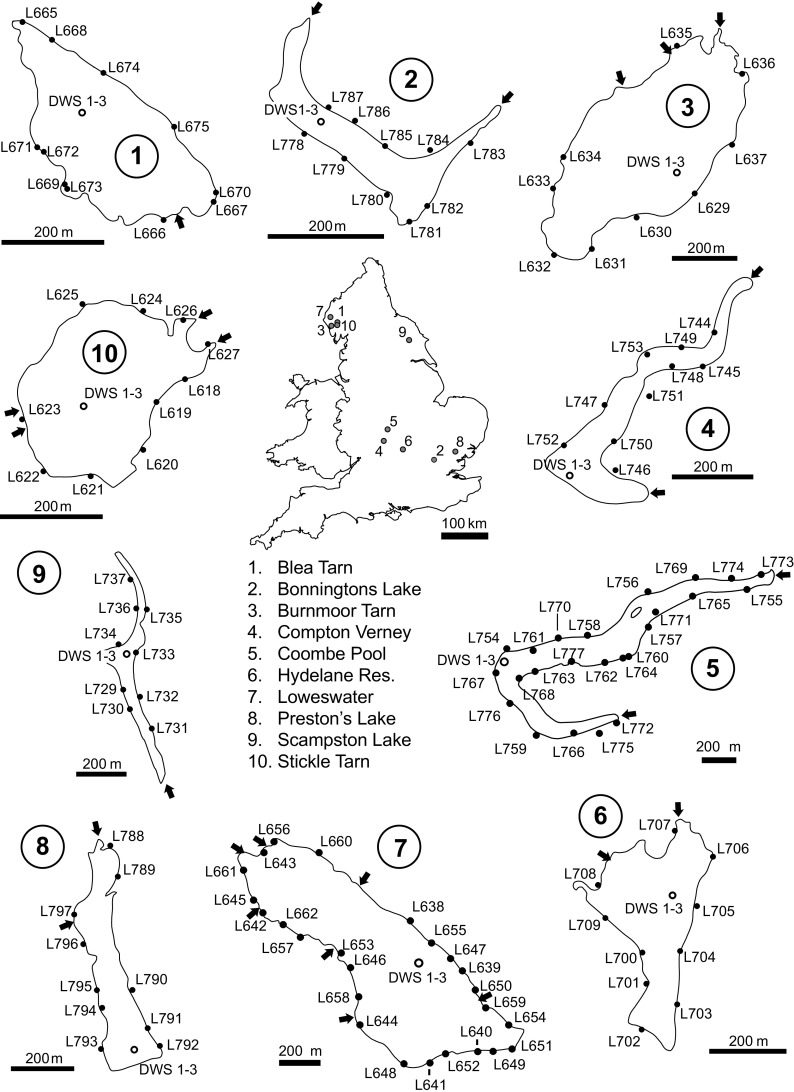
Location of calibration lakes in England with littoral (*L*) and deep water (*DWS*) sediment sampling locations. Soil and inflow sample locations are in Online Resources

In order to assess how littoral sediment samples (a) vary around a lake and (b) compare to deep water sediments, inflowing stream sediments and catchment soils from which they may be at least partially derived, ten lakes were selected in England for detailed study. These were divided between upland and lowland sites and represent a range of catchments and lake types (Table 1 and Fig. 1) (see site descriptions in Online Resource 1). Sites were chosen by availability of catchment soil and inflow stream sediment metals data in G-BASE and archived sediments available for Hg measurement. Additional soil samples were nonetheless required and collected for Loweswater, Blea Tarn, Burnmoor and Stickle Tarn in the Lake District. At these upland sites, G-BASE protocols were followed with the collection and analysis of both surface peat (A-horizon, 5–20 cm) and a lower depth (S-horizon, 20-50 cm) when required (Johnson 2005). For the other lowland lakes, soil samples are from the top 5–20 cm of soil. At Bonnington’s Lake and Stickle Tarn, stream sediments were unavailable.

**Table 1:** Calibration water body location and summary information

Name	WBID	Latitude	Longitude	Altitude (m)	Area (ha)	Max. depth (m)	Perim. (km)	Max fetch (m)	SDI	Catchment area (ha)
Blea Tarn	29097	54° 31′ 02″	03° 05′ 44″	478^a^	7.4	12.0	1.2	510	1.247	128.2
Bonnington’s Lake	40634	51° 47′ 59″	00° 42′ 37″ E	57	2.8	1.8	1.2	375	1.944	337.7
Burnmoor Tarn	29215	54° 25′ 46″	03° 15′ 28″	253^a^	23.9	13.0	2.2	857	1.280	625.5
Compton Verney	39036	52° 10′ 07″	01° 33′ 04″	78	13.1	2.0	3.6	1200	2.836	923.0
Coombe Pool	37926	52° 24′ 43″	01° 25′ 15″	73	30.6	2.0	5.9	1500	2.987	4402.5
Hydelane Lake	39750	52° 00′ 37″	00° 56′ 40″	74	11.4	2.5	2.6	646	2.149	570.5
Loweswater	28986	54° 34′ 52″	03° 21′ 19″	125^a^	60.3	16.5	3.9	1700	1.432	818.5
Preston’s Lake	n/a	51° 57′ 22″	00° 41′ 51″ E	51	7.7	3.5	1.9	744	1.890	1270.0
Scampston Lake	29682	54° 09′ 53″	00° 40′ 27″	32	4.9	1.2	2.1	587	2.627	122.0
Stickle Tarn	29177	54° 27′ 28″	03° 06′ 16″	473^a^	7.4	12.5	1.1	395	1.140	197.0

Morphometric data from UK Lakes Portal (https://eip.ceh.ac.uk/apps/lakes/index.html) and fieldwork (max depth)

^a^UK upland catchments

Three cores were collected at the same location from the deepest area of the lake using a HTH gravity corer (Renberg and Hansson 2008), and the surface sediments of each core (0–1 cm) were retained. At 8 of the 10 sites, 10 littoral sediment samples were taken approximately equally spaced from around the full perimeter of the lake, using a stainless steel ladle from the shoreline as suggested in the metals survey instructions. At two sites (Loweswater and Coombe Pool), more samples were taken in order to try and sample each littoral location that a participant in the survey could easily access. This resulted in 25 littoral samples from Loweswater and 24 littoral samples from Coombe Pool. All calibration littoral and surface samples were treated as described above for the metals survey samples. Archived soil and stream sediments used had all been previously sampled using G-BASE field protocols (Johnson 2005).

### Geochemical methods

Sediment processing followed the protocol developed by the BGS for G-BASE (Ault 2006). Briefly, wet sediments were washed through a 2-mm sieve using distilled water to remove large detritus and then through a 150-μm sieve. After settling, the <150-μm fraction was freeze-dried and milled using an agate pestle and mortar (timed 3 min).

Weighed (4 d.p.) mixtures of freeze-dried milled sediment (∼2 g) and binding wax (0.9 g) (Licomax C, Hoechstwax Flux) were placed in a pneumatic press (15 t). Batches of pressed pellets were measured in an X-ray fluorescence spectrophotometer (Spectro-X Lab 2000) with a reference sediment (CANMET LKSD-2) pellet of similar mass in each run. Recovery rates between the measured and reported element values for the reference material in the sample batches were Ni (94%), Cu (105%), Zn (106%) and Pb (99%) (Online Resource 2).

For Hg, subsamples of sieved and freeze-dried sediment were digested with 8-ml aqua regia at 100 °C on a hotplate for 2 h in acid-leached 50-ml polypropylene digestion tubes. Digested solutions were analysed for Hg using cold vapour-atomic fluorescence spectrometry (CV-AFS, Millennium Merlin 1631 system) following reduction with SnCl_2_. Standard solutions and quality control blanks were measured every five Hg samples to monitor measurement stability. Reference stream sediment GBW07305 (Shanghai Institute of Nuclear Research, certified Hg value 100 ± 10 ng g^−1^) and sample blanks were digested with every 20 samples. A mean value of 106 ng g^−1^ for the reference sediment samples was recorded during analysis of the OPAL samples. Soil samples were processed using the same method. As none of the archived soil data on G-BASE included Hg concentrations, samples of catchment soils and inflow stream sediments available from the BGS archive were additionally analysed for Hg with sediment samples. The organic contents of littoral, benthic and stream sediment samples were measured gravimetrically by loss-on-ignition (LOI) (2 h, 550 °C; Heiri et al. 2001). Organic contents of G-BASE soil samples were determined by heating for 4 h at 450 °C (Ault 2006). Organic matter (LOI) calculated by using this lower temperature and longer duration may only vary c. 2% (Matthiessen et al. 2005) in organic soils.

### Inter-laboratory comparison

As archive data from G-BASE, as well as from new analyses, were used for the calibration lakes, an inter-laboratory comparison (ILC) was undertaken at UCL using two stream and five soil samples previously analysed and available from the BGS archive. These samples were prepared and analysed by XRF at UCL using the method employed for the metals survey samples. Reference sediment (LKSD2) was also processed and measured during the ILC. For the ILC stream sediment samples, the difference between the BGS and UCL measurements for Ni, Cu, Zn and Pb was 1.5, 0.2–1, 0.2–1.8 and 1.7–3.5 μg g^−1^, respectively (Online Resource 1). There was more variability in the soil samples between 1.1 and 1.5, 3.5 and 7.7, 5.2 and 33.8 and 0.3 and 23.1 for Ni, Cu, Zn and Pb, respectively. Within sample variability, rather than significant differences due to BGS/UCL methodologies, is indicated by less variability in concentrations between UCL measured and reported values for the reference sediment during the ILC calibration runs (Ni 1.8, Cu 1.9, Zn 7.1 and Pb 0.4 μg g^−1^, respectively).

### Data analysis and interpretation

The MS EXCEL Add-in for Robust Statistics (http://www.rsc.org/Membership/Networking/InterestGroups/Analytical/AMC/Software/RobustStatistics.asp) was used to calculate Median Absolute Deviation (MAD), Huber’s mean (h^15^ *x̄*) and standard deviation. Unlike an arithmetic mean (average), MAD is not sensitive to outliers. Hubers mean similarly is a more robust estimate of the average (AMC 2001). A Geographical Information System (GIS) was used (ArcMap 10.2.1) for visual exploration of the data and spatial mapping. Low-dimensional plots (PC Axis 1 vs. 2) from Principal Component Analysis (PCA) were calculated (IBM SPSS 22) to aid in identification of outlying values and clusters of similar observations (Online Resource 1). Element concentrations were standardised and log transformed to a parametric distribution. The percentage difference (%*D*), the absolute value of the difference between two samples over their mean multiplied by 100, was used to determine the variation between individual littoral samples around the lake.

Sediment quality guidelines have been developed that allow an assessment of potential risk to aquatic biota based on the concentration of elements and other compounds. Elevated metal concentrations in lake and pond sediments have the potential to affect freshwater ecosystems by food chain uptake, via sediment-dwelling (benthic) organisms and re-mobilisation of metals across the sediment water interface by biogeochemical processes. The OPAL Metals Survey uses a consensus-based three-tier classification of effect concentrations (MacDonald et al. 2000) for Hg, Ni, Cu, Zn and Pb. Concentrations below a Threshold Effect Level (<TEC) indicate that harmful effects are unlikely to be observed, whereas concentrations at and above a Probable Effect Level (PEC) suggest harmful effects are likely to be observed due to that element alone. An assessment of sediment quality by evaluating the effect of the mixture of the five metals was also calculated by calculating mean PEC quotients (mPEC-Q) (MacDonald et al. 2000).

## Results and discussion

This section is structured to show the variability of metal elements in littoral lake sediments and a comparison of littoral sediments with deep water, inflow stream sediment and local soils. Results are discussed from the calibration lakes to show that littoral sediments can provide a baseline measure of sediment contamination and potential toxicity in a water body. This knowledge is then applied to the results of the national-scale snapshot of sediment metal concentrations in English ponds and lakes aided by public participation.

### Variability of element concentrations in littoral sediments of the calibration lakes

**Fig. 2:**
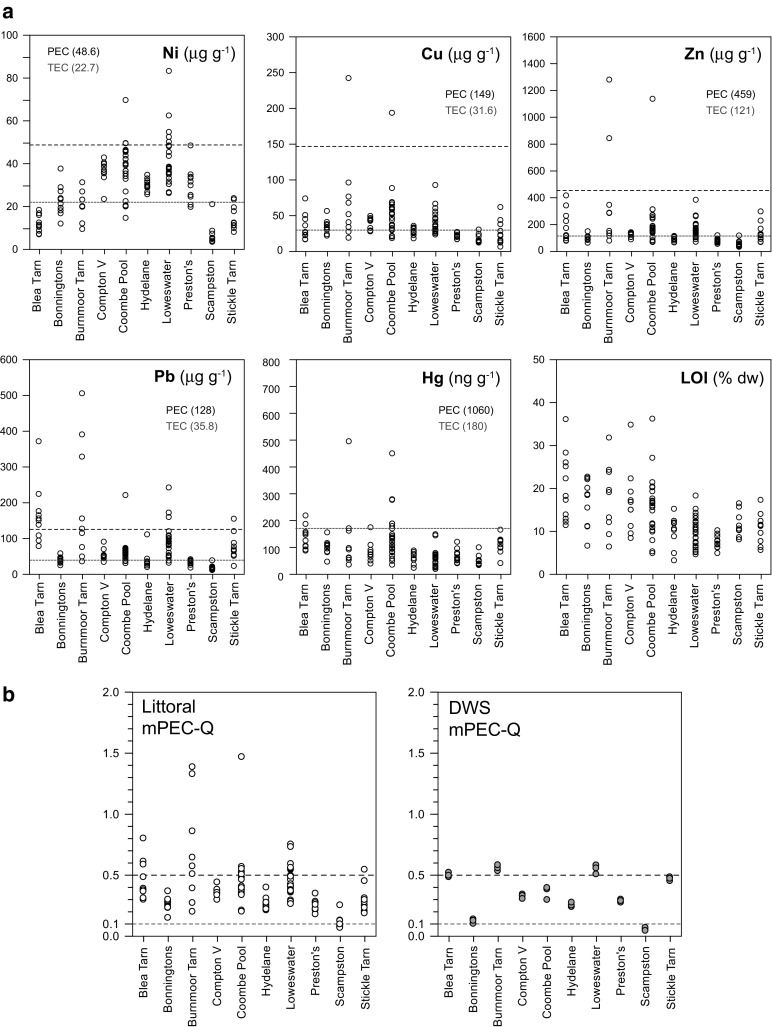
**a** Metal concentrations and LOI of littoral samples from the OPAL calibration lakes. Consensus-based PEC and TEC values (MacDonald et al. 2000) are also shown for each metal element. **b** Mean Probable Effect Concentrations (mPEC-Q) for littoral (*left*) and deep water sediments (*DWS*) in the calibration lakes; mPEC-Q calculated with Hg, Ni, Cu, Pb and Zn. Sediment samples predicted to be not toxic where mPEC-Q <0.1 or <0.5 (MacDonald et al. 2000)

Concentrations of Hg, Zn, Cu, Pb, Ni and LOI values found in the littoral samples at each lake are shown in Fig. 2. Sampling locations, codes and concentrations for each metal at the calibration lakes can be found in Online Resources 3 and 4, respectively.

Differences in littoral element values vary between and within lakes of the calibration dataset (Fig. 2), with outliers caused by localised high concentrations (Online Resource 4; see also Online Resource 3 for sample codes). For each of the determinants, comparison of the %*D* between littoral values and with the lake mean littoral (h^15^ *x̄* LS) show sampling location and organic composition are controls (Online Resource 5). In Burnmoor Tarn, differences (reduced %*D*) look to be due to the proximity of inflows but also due to shoreline characteristics (sheltered vs. open) that influence fine-grained/organic sedimentation. In Stickle Tarn, samples near inflows also vary: at sample L623, there was less organic matter (and Hg) with a greater %*D* in the other metals. Near the main inflow (NE corner), the locally sheltered position increases organic matter and all metals in sample L626. By contrast, L621 has markedly lower than mean littoral values in metals (except Ni) due probably to its exposed position (less organic matter) and proximity to a low concrete dam wall. In Blea Tarn, small differences compared to the mean (h^15^ *x̄* LS) in littoral organic composition result in higher (L669) and lower (L673) metal concentrations. At Loweswater, local organic content (determined by shoreline aspect) also appears more of a factor than proximity to inflow.

In the larger lowland lakes (Table 1 and, Online Resource 5), a more complex pattern is observed between littoral samples due to organic matter content, lake situation and small scale variations in mineral-metal content. In Coombe Pool, significant %*D* between littoral sediments occurs in samples that are ∼3× less than mean LOI on opposite sides of the central part of this sinuous lake (L771, L757-8). The outlier (L775) with elevated metal concentrations and organic matter in the southeast arm of the lake is evident.

In the smaller lowland lakes, %*D* varies less. In Bonnington’s Lake, samples L782–L786 and Hydelane Reservoir L702–707 and L709 show limited %*D* of littoral values. Significant differences do occur, as in the upland lakes, though these appear less related to catchment inputs and more related to the local distribution and contamination of sediment within the littoral zone. In Hydelane Reservoir for example, Pb values at L700 (109.8 μg g^−1^) is ∼+100%*D* from the lake mean compared to adjacent L701 (18.4 μg g^−1^, −50%*D*).

### Differences between LS, DWS, soils and inflow stream sediments

Comparison of littoral (LS), deep water (DWS), soil and inflow stream sediments provides further data on using LS to assess lake-wide metal values. Online Resource 6 shows the differences in concentrations and %*D* for the calibration lakes between the sediment/soil compartments. Variation between soils, streams and lake sediments is expected due to inherent differences in environmental samples (e.g. stream sediments being coarser grained and less organic than littoral sediments) and spatial heterogeneity of soil/sediment compartments between studied lake catchments.

The type of soil and its transport and deposition to DWS are illustrated in the upland lakes, where metal-enriched peat soils occur (Rose et al. 2012). Stream and littoral sediment concentrations are a mixture of eroded peat and mineral sediments due to their more dynamic depositional environments, while deep lake sediment concentrations receive eroded peat directly and reworked littoral and pelagic organic matter. Differences in Pb concentrations between surface peat (162–291 μg g^−1^) and deep soil (10.1 μg g^−1^) and comparably high Pb concentrations in stream sediment, littoral and pelagic sediments suggest atmospherically contaminated peat is a principal source of Pb to Blea Tarn. The organic content of the littoral (h^15^ *x̄* = 19.8%) and pelagic sediments (h^15^ *x̄* = 37.5%) compared to the surface peat samples (LOI 83–95%) indicates eroded peat forms only a fraction of littoral sediment. Zn concentrations have a greater range than the other metals in the littoral zones, due perhaps to mixing of both peat and soil-mineral sources. Cu and Ni are comparatively low and uniform. The influence of surface peat-containing metal elements is evident in the PCA axis scores, with the littoral and benthic sediments contrasting with a below-peat mineral soil sample (Online Resource S3a, marked S1).

At Burnmoor Tarn, high Hg (493.2 ng g^−1^) and Zn (1276 μg g^−1^) were found in one littoral sample from the south of the lake. Pb concentrations are elevated (>100 μg g^−1^) in most of the DWS, LS, inflow streams and surface soils. Similar to Blea Tarn, surface organic peat soil concentrations of Pb are higher than deeper soils, as a likely result of modern industrial atmospheric contamination. Also like Blea Tarn, concentrations of Cu and Ni are low in Burnmoor except for a littoral hotspot of Cu at L629 (241 μg g^−1^). In Stickle Tarn, metals are again elevated (×2–×4 compared to deeper soil) in the surface peat samples and in the deep lake sediment, with distinct separation of the sediment compartments observed in the PCA for the five metals and LOI. Elevated Ni values are distributed around Loweswater with the highest at the shallow ends of the lake, perhaps indicating concentration of Ni by wave activity. Highest Pb concentrations occur at the lake margin of wooded alluvial fans that abut the south-southwest edge of the lake, suggesting a catchment-derived mineral source. Bedrock concentrations of Pb (approximately 150 μg g^−1^) are found at nearby Bassenthwaite Lake, which like the geology west/south west of Loweswater is on the Skiddaw Group (Ineson 1994) that has been mined historically for Pb, Zn and other metals. Littoral Cu and Zn values are generally low, except for a single high Zn value of 379 μg g^−1^ associated with more organic sediment. Littoral and DWS sediment Hg and Zn concentrations show a positive correlation with organic matter (*r* ^2^ = 0.5 and 0.63, respectively).

In the lowland lakes, element concentrations between soil/sediment compartments are more similar. primarily, we suggest, to the nature of soil in the catchment and absence of peat/organic soils. Lower values of Hg, Ni, Zn and Pb in LS and DWS at Hydelane Reservoir and Scampston Lake, compared to of soil and stream sediments, suggest a disconnect of sediment transport between the lake and catchment. In Bonnington’s Lake, littoral, soil, stream and central lake sediments are similar. Concentrations of LS metals at Bonnington’s Lake are higher in almost all samples than the DWS samples, likely due to a greater organic (macrophyte origin) and fine sediment content in the margins. In Compton Verney, littoral Ni concentrations appear uniformly enhanced (h^15^ *x̄* = 37.4, SD = 3.8, MAD = 2.4 μg g^−1^) but are comparable to catchment soils (*x̄ =* 42.5) and inflow stream sediments (*x̄ =* 45.7). Like Ni, the concentrations of Pb and Cu vary little (MAD = 2–2.6 μg g^−1^), whereas Zn and Hg are more variable (MAD = 8.5–13). Concentrations of Cu, Pb and Hg show some correlation with LOI (*r* ^2^ = 0.5–0.7) but LOI values are low, no higher than 20% (h^15^ *x̄ =* 15.8, SD = 5.9). Although a complex water body shape, littoral and pelagic surface concentrations of Hg, Zn, Cu and Pb in Coombe Pool are comparable but slightly higher in the littoral samples. There is a strong relationship between values of LOI with Cu (*r* ^2^ = 0.9) and with Zn (*r* ^2^ = 0.65) (Table 2a). The outlier value of Cu, Pb, Zn and Hg at the end of the southern arm of the lake is likely due to urban soil or dredged lake mud dumped near the shoreline.

**Table 2:** Matrix of correlation coefficients (*r* ^2^) between metals and LOI (organic content) and metals and titanium (mineral content) for littoral sediments

Calib. lakes^a^		Ni	Cu	Zn	Pb	Hg
Blea	LOI	0.02	0.20	0.04	0.05	0.06
	Ti	0.00	**0.62**	0.15	0.02	0.04
Bonn	LOI	0.02	0.13	0.24	0.05	0.01
	Ti	0.55	0.03	0.01	0.00	0.17
Burn	LOI	0.23	0.37	0.01	0.01	0.02
	Ti	0.02	**0.62**	0.12	0.06	0.17
ComptV	LOI	0.04	**0.60**	0.35	0.57	**0.68**
	Ti	0.54	0.59	0.29	0.12	0.11
CoombeP	LOI	**0.63**	**0.84**	**0.67**	0.50	0.52
	Ti	0.03	0.13	0.11	0.09	0.00
Hydelane	LOI	0.01	0.54	0.53	0.35	0.47
	Ti	0.09	0.34	0.18	0.16	0.35
Loweswater	LOI	0.48	0.01	0.61	0.01	0.23
	Ti	0.07	0.03	0.16	0.03	0.01
Preston’s	LOI	0.01	0.47	0.47	0.28	0.26
	Ti	0.34	0.09	0.41	**0.70**	0.17
Scampston	LOI	0.49	**0.64**	0.40	0.48	**0.66**
	Ti	**0.76**	0.47	**0.92**	**0.85**	**0.80**
Stickle	LOI	0.02	0.00	0.00	0.03	0.43
	Ti	0.59	0.16	0.57	0.40	0.00
Regions^b^		Ni	Cu	Zn	Pb	Hg
East England	LOI	0.03	0.52	0.30	0.15	0.58
	Ti	0.29	−0.16	0.02	0.04	0.11
East Midlands	LOI	−0.08	0.44	0.35	−0.03	0.03
	Ti	0.41	−0.22	−0.10	0.09	−0.10
London	LOI	0.38	0.57	**0.61**	**0.82**	**0.98**
	Ti	−0.23	−0.61	−0.56	−0.92	−0.96
North East	LOI	0.08	**0.93**	**0.87**	−0.08	0.55
	Ti	0.31	−0.44	−0.33	−0.18	−0.40
North West	LOI	−0.10	0.26	0.25	0.26	0.05
	Ti	0.29	−0.12	−0.16	−0.06	0.02
South East	LOI	0.28	0.33	0.36	0.28	0.13
	Ti	−0.08	−0.21	−0.22	−0.08	−0.04
South West	LOI	0.04	−0.20	0.26	−0.07	0.04
	Ti	−0.22	−0.33	−0.60	−0.33	0.02
West Midlands	LOI	−0.17	0.21	0.25	0.25	0.09
	Ti	0.18	−0.22	−0.34	−0.33	−0.26
Yorks and Humber	LOI	−0.09	**0.68**	0.51	0.36	0.53
	Ti	0.08	−0.21	−0.37	−0.58	−0.61
ALL	LOI	−0.03	0.19	0.29	0.19	0.12
	Ti	0.18	−0.09	−0.13	−0.09	−0.07

Values in bold = *r* ^2^ > 0.6

^a^Calibration sites

^b^National survey by regions and England

The low organic contents of deep benthic sediments (LOI *x̄* = 7.3, SD = 0.17) in Hydelane are comparable to littoral (h^15^ *x̄* = 10.3, SD = 3.5) samples. The maximum Pb concentration (109.8 μg g^−1^) occurred in a sample collected from an angling swim, where discarded lead fishing weights may be a source. Concentrations of Hg, Ni, Pb, Cu and Zn are low in Preston’s Lake, with soils, inflow stream sediment and benthic sediments all comparable. Only nickel appears elevated with a maximum littoral sediment value of 48.2 μg g^−1^. Similarly, metal concentrations are low in all Scampston Lake sediments and surrounding soils and streams. Littoral values of Cu, Ni and Pb differ little (MAD = 1.2–2.3). Hg values poorly correlate with LOI content (*r* ^2^ = 0.4), while Zn appears to vary with mineral matter based on its correlation with titanium (Ti) (Table 2a).

Preferential deposition of organic matter and likely transport from shallow to deep water are evident in Stickle Tarn, Loweswater and Blea Tarn (−78, −62 and −61.5%*D* in LOI between LS and DWS, respectively). This exposed shoreline process also occurs in Burnmoor Tarn, although locally sheltered littoral areas with higher organic matter content reduce the difference (32.3%*D*). A similar small difference occurs in Preston’s Lake (35.6%*D*), with slightly lower organic sediment content (*x̄* = 7.4%) around the margin of this small lake, than the deep area (10.6%). Conversely, LS organic matter values are higher than DWS in the lowland lakes due presumably to the proximity of abundant marginal aquatic vegetation and lower-energy shoreline dynamics. However, the actual difference between mean LS and DWS LOI values is small (3–7%).

Adsorption of metals to organic matter influences differences in concentrations between LS and DWS. DWS concentrations of Hg in Blea, Burnmoor, Loweswater and Stickle are much higher: 60–99%*D* and 90–140 ng g^−1^ (Online Resource S6). In Bonnington’s Lake and Scampston Lake, Hg values are 40–50%*D* and 19–33 ng g^−1^ lower. Differences between LS and DWS concentrations of Cu, Zn and Pb are likely influenced by their attachment to organic matter but complicated by mixing with mineral matter. Ni is almost consistently lower in DWS samples than in LS that point to its association with inorganic, mineral littoral sediments (e.g. Scampston Lake, Table 2a).

### Potential toxicity of soils and sediments to aquatic biota in the calibration dataset

Translation of soil and sediment concentration values for individual elements to numerical boundaries of TEC and PEC sediment quality guideline (SQG) values (MacDonald et al. 2000) are useful for within and between lake comparison and highlighting local hotspots (Fig. 2 and Online Resource 4a–j). The grey (TEC) and black (PEC) shadings in Table 3 smoothen the variability found at each lake but highlights similarities and a broad assessment of potential metal toxicity to aquatic biota. Deep water, more organic surface sediment metal values appear consistently higher than littoral sediments in the upland lakes. This is consistent with the preference of the measured metals with fine-grained and organic matter (due to adsorption, large surface area) and known movement of peat contaminated by historical atmospheric deposition into UK upland lakes. In the smaller, lowland lakes, TEC/PEC differences between DWS and LS are comparable/lower due to less difference in organic matter and potential lake-wide mixing due to shallowness. This lake type/peat-dependent LS/DWS difference is highlighted with the calculated range of mPEC-Qs for LS and DWS (Fig. 2b).

**Table 3:**
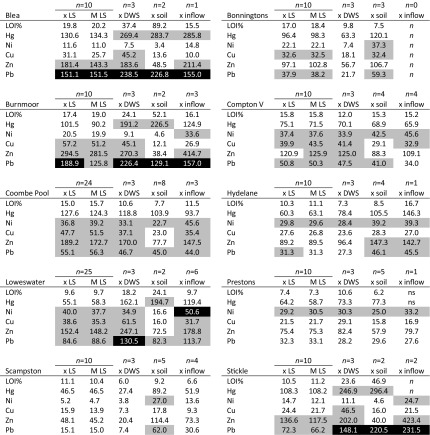
Application of >TEC (grey) and >PEC (black) categories of metal concentrations to values found in mean (H15) of littorals (xLS), median of littorals (M LS), mean of deep water sediments (xDWS) and mean soil and inflow samples

Mean of DWS, soil and inflow arithmetic mean due to small sample number. No shading < TEC. *n* = no. of sample

In Blea Tarn, concentrations of Pb in 7 out of 10 of the littoral and benthic sediments are above PEC concentrations. Other metals reach TEC concentrations but Pb is clearly the major factor in the potentially toxic mPEC-Q measured in the lake. Greater than TEC and PEC concentrations of Hg, Ni, Zn, Cu and Pb occur in Burnmoor. All littoral samples have an mPEC-Q >1, with six out of nine samples >0.5 and two >1, while the mPEC-Q of DWS are >0.5. Although industrial atmospheric deposition has likely contributed to potential toxic concentrations in Burnmoor, the two littoral samples with mPEC-Qs of 1.3–1.4 (L631 and L632) occur at the foot of the slope below a nineteenth century shooting lodge. The mPEC-Qs of inflowing Burnmoor stream sediments (0.3, 0.4. 1.1) also indicate catchment-scale metal contamination. TECs of Ni, Cu, Zn and Pb occur in Loweswater, while 7 of the 25 littoral samples and DWS have an mPEC-Q of >0.5. In Stickle Tarn, Pb shows littoral concentrations >TEC in nine of the samples with one >PEC value, while DWS mPEC-Q values are near 0.5 (0.46–0.49). Near to or above littoral TEC values are seen in Compton Verney for Ni, Cu, Zn and Pb that generates mPEC-Qs >1. In Coombe Pool, all littoral samples have an mPEC-Q >0.1, while 7 out of 24 samples have an mPEC-Q >0.5, including the highest mPEC-Q calculated for the calibration lakes at L775 (1.47).

### Calibration summary

**Fig. 3:**
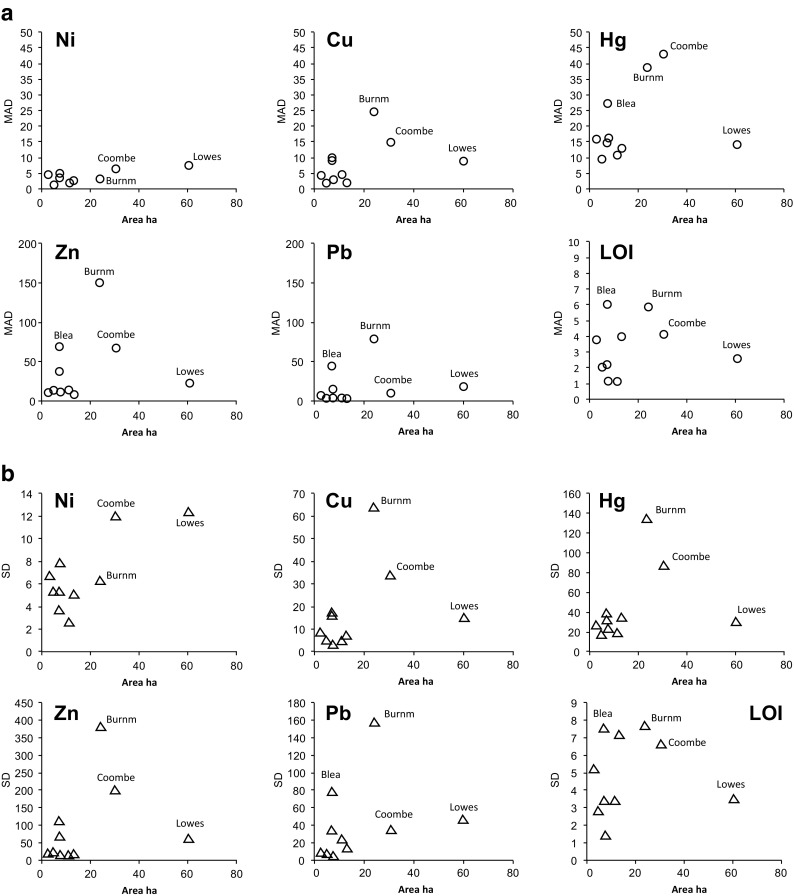
**a** MAD and **b** SD element values of littoral sediment samples vs. lake area

In the calibration lake dataset therefore, absolute differences between littoral sediments around a lake are not large (except hotspot outliers) (Fig. 2). The dataset indicates if a lake is small (<20 ha) and the MAD and standard deviation of littoral samples are low (Fig. 3). With increased size and complexity of inputs and diversity of lake habitats, upland or lowland sources, the dataset littoral sediment concentrations are more varied. TEC/PEC and MPEC-Qs calculations suggest that a littoral sample can be comparable to deep water, central surface samples, especially in smaller, lowland lakes and ponds.

## OPAL Metals Survey results

In 2010, 395 OPAL Metals Survey packs were requested and 120 samples from individual water bodies were returned representing a 30% return. One hundred fifty other single lake samples were collected by student and research groups conducting fieldwork during the survey period, using the same protocols. Single samples from individual lakes and ponds (*n* = 41) were also collected during fieldwork for the OPAL Water Centre Aquatic Monitoring Programme (Turner et al. 2013) and at OPAL public engagement events. The calibration exercise generated 129 littoral samples from 10 lakes across England, although only the first sample collected at each of the lakes was entered, to replicate how it would have been included if collected by a survey participant. The ‘first’ sample was determined upon arrival at the lake and the first location where a suitable sampling location that matched the suggestions/guidance given in the survey pack. We were not expecting participants to survey the whole lake margin before selecting a sample point nor have the hindsight of prior measurements. Using the first sample collected reduces selective bias and maintains the semi-random nature of the sampling, as compared to using the sample with highest metal concentrations to represent the calibration lake in the public participation survey dataset.

**Fig. 4:**
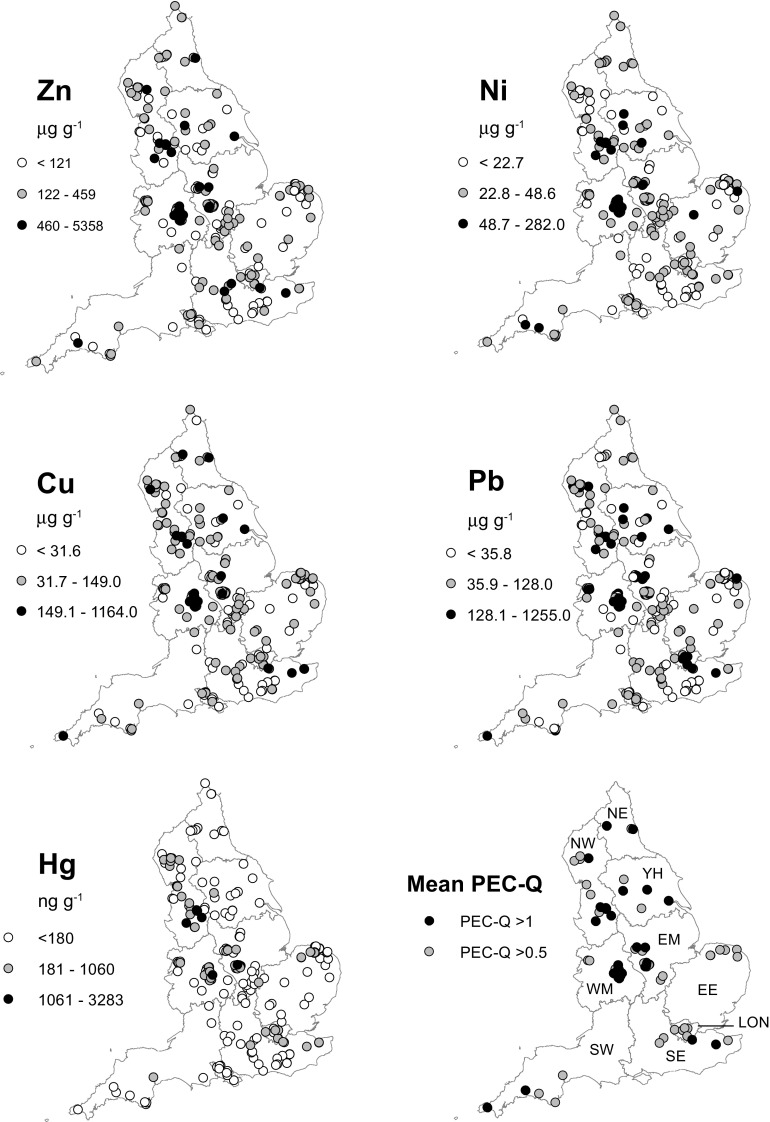
Distribution map of element concentrations in pond and lake sediments sampled by OPAL Metals Survey participants 2010. Element values categorised by consensus-based sediment quality guidelines: <TEC (*white*), >TEC (*grey*) and >PEC (*black*). *Bottom right*: mean PEC quotients (>0.5 and >1) for Ni, Cu, Zn, Pb and Hg (MacDonald et al. 2000). Boundaries and abbreviations of English regions are shown

Littoral sediment element concentrations from 291 water bodies were analysed by XRF, 236 for Hg. The dataset is a spatially random (with local clustering) dataset of water bodies across England (Fig. 4). Clusters of sites exist where local interest directed sampling, e.g. in Birmingham and north Norfolk. The location data provided by participants allowed the dimensions of 269 water bodies to be measured using Google Earth Pro™. Twenty-seven lakes were not found, due to tree cover or multiple water bodies in the vicinity and lack of detail of location data. A polygon drawn around visible open water was used to calculate the area of identified water bodies. Of these, 261 sites were <50 ha (97%), 253 <20 ha (95%) and 247 <10 ha (95%). The mean area of identified lakes was 10.3 ha (median = 0.13 ha).

**Fig. 5:**
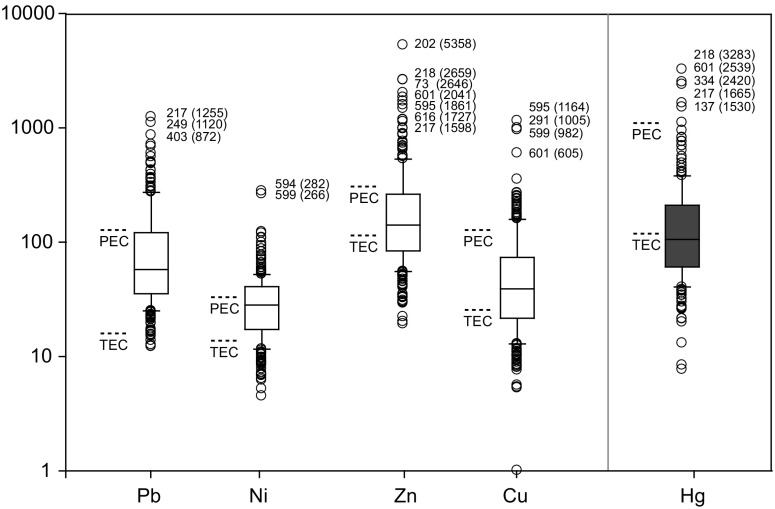
Box and whisker plot of OPAL national survey results for Hg, Ni, Cu, Zn and Pb. Log scale to show detail of outliers. Ni, Cu, Zn, Pb in micrograms per gram. Hg values are in nanograms per gram. Three-figure numbers are OPAL sample codes (Table 5). Consensus-based TEC and PEC concentrations are shown for each element as *dashed lines*. Whisker extents are 10th and 90th percentiles, respectively. *Box* extent represents 50% of the data

Although the OPAL Metals Survey was devised and planned nationally, as with the other OPAL surveys, communication and encouragement to participate were managed by scientists from nine regional universities (Davies et al. 2011). The nomenclature and geocoding of the nine regions correspond to the Eurostat NUTS1 boundaries of England (Table 4). Breaking down the data into these regions, there is a spatial bias of numbers of samples from East of England (23%), North West England (16%), West Midlands (15%), East Midlands (15%) and South East England (15%) compared to the South West, Yorks and Humber and North East England (4–5%) and London (1.7%). Significantly elevated concentrations of Pb, Zn, Cu and Ni were more prevalent in samples from North West England and West and East Midlands, though mapping shows clearly that >PEC values occur in all regions (Table 5). Compared to the other metals, Hg contamination is not widespread across England. Significantly high concentrations of Hg appear to be clustered in North West England and the West Midlands (Fig. 4 and Table 5). In London, the number of lakes sampled was very low and almost certainly masks the true extent of metal contaminated water bodies in the London region (Hall 2013).

**Table 4:** Regional and national summary of of metal concentrations from public participant samples

NW (*n* = 48)	Min	Max	*M*	MAD	H15 [SD]	NE (*n* = 13)	Min	Max	*M*	MAD	H15 [SD]
Ni	7.8	92.9	30.2	13.6	31.4 [18.7]	Ni	8.4	47.2	28.7	7.3	28.4 [13.2]
Cu	13	255.6	46.0	24.6	65.6 [49.6]	Cu	5.6	255.5	35.1	22.4	44.0 [35.5]
Zn	29.3	5358.0	132.7	53.3	163.7 [111.]	Zn	33.1	2646.0	146.1	124.0	156.8 [82.1]
Pb	19.8	1255.0	86.9	48.9	133.9 [127.3]	Pb	12.7	164.7	56.3	28.0	56.5 [35.4]
Hg (*n* = 44)	8.4	3283.0	169.5	115.1	238.1 [235]	Hg (*n* = 9)	47.8	179.6	97.8	44.4	100.9 [56.7]
WM (*n* = 44)	Min	Max	M	MAD	H15 [SD]	EM (*n* = 44)	Min	Max	M	MAD	H15 [SD]
Ni	15.1	282	42.5	9.7	44.6 [18.6]	Ni	12.4	82.1	36.0	9.8	34.8 [12.5]
Cu	16.9	1164	119.1	67.9	131.7 [95.2]	Cu	5.4	268.8	43.2	17.5	47.3 [30.1]
Zn	81.9	2041	373.5	187.0	413.7 [284.8]	Zn	45.6	1489.0	203.5	112.3	228.8 [165.2]
Pb	15.4	720	160.4	102.7	161.3 [121.0]	Pb	21	1120.0	58.1	22.4	78.1 [59.7]
Hg (*n* = 35)	20	2539.7	242.8	140.6	250.7 [190]	Hg (*n* = 40)	21.3	2420.7	111.4	61.3	126.1 [89.1]
SW (*n* = 12)	Min	Max	M	MAD	H15 [SD]	LON (*n* = 5)	Min	Max	M	MAD	H15 [SD]
Ni	6.4	120.0	37.8	11.4	35.2 [22.6]	Ni	5.2	46.6	25.6	–	–
Cu	1	1005.0	31.7	14.5	41.3 [33.8]	Cu	10.1	94.1	69.7	–	–
Zn	22.3	563.9	131.5	66.8	160.3 [118.7]	Zn	47.3	284.0	238.4	–	–
Pb	15	281.3	52.5	31.0	67.7 [48.1]	Pb	83.5	189.1	158.4	–	–
Hg (*n* = 10)	48.22	216.5	128.3	39.3	116.5 [51.8]	Hg (*n* = 4)	57.6	441.4	296.2	–	–
YH (*n* = 14)	Min	Max	M	MAD	H15 [SD]	EE (*n* = 67)	Min	Max	M	MAD	H15 [SD]
Ni	4.5	110.1	36.9	13.1	40.1 [21.7]	Ni	6.2	108.9	19.3	5.8	19.9 [8.2]
Cu	14.3	167.1	45.2	20.3	57.0 [42.7]	Cu	5.3	95.8	26.3	10.2	27.2 [14.4]
Zn	34.8	1002.0	162.0	90.3	214.3 [175.0]	Zn	35.0	379.0	105.0	33.9	110.3 [52.3]
Pb	12.3	872.0	90.6	58.1	106.7 [91.6]	Pb	12.2	260.6	40.2	10.8	44.9 [19.3]
Hg (*n* = 13)	7.7	466.7	100.5	39.4	89.0 [64.7]	Hg (*n* = 45)	29.9	344.3	93.8	37.8	103.6 [56.7]
SE (*n* = 44)	Min	Max	M	MAD	H15 [SD]	England (*n* = 291)	Min	Max	M	MAD	H15 [SD]
Ni	6.8	48.6	19.9	7.8	20.6 [10.2]	Ni	4.5	282	28.3	11.6	28.7 [16.4]
Cu	7.6	201.0	26.6	13.5	28.5 [18.6]	Cu	5.3	1164	39.2	19.7	47.7 [37.3]
Zn	19.3	766.0	97.0	54.7	114.4 [81.7]	Zn	19.3	5385	142.0	68.5	175.4 [134.5]
Pb	15.6	487.7	42.5	21.3	53.0 [33.6]	Pb	12.2	1255	57.9	27.7	77.5 [61.6]
Hg (*n* = 36)	26.3	955.9	74.4	28.2	85.7 [52.8]	Hg (*n* = 236) ng g^−1^	7.7	3283	106.2	56.3	104.6 [102.8]

All values in micrograms per gram, except for Hg in nanograms per gram. Abbreviations of the regions correspond to the Eurostat NUTS1 boundaries of England. Minimum, maximum, median (*M*), Median Absolute Deviation (MAD) and mean H15 and H15 standard deviation

>*NW* North West, *WM* West Midlands, *SW* South West, *YH* Yorkshire and Humber, *SE* South East, *NE* North East, *EM* East Midlands, *LON* London, *EE* East of England (http://neuropa.eu/eurostat/documents/345175/7451602/nuts-map-UK.pdf)

**Table 5:** Number of sites >TEC and >PEC exceedance by region

NW (*n* = 48)	>TEC	>PEC	NE (*n* = 13)	>TEC	>PEC
Ni	30 (62.5)	10 (20.8)	Ni	10 (77)	0 (0)
Cu	37 (77.1)	7 (14.6)	Cu	8 (61.5)	2 (15.4)
Zn	26 (54.2)	8 (16.6)	Zn	10 (76.9)	1 (7.7)
Pb	41 (85.4)	17 (35.4)	Pb	9 (69.2)	1 (7.7)
Hg (*n* = 44)	22 (5)	4 (9.1)	Hg (*n* = 9)	0	0
WM (*n* = 44)	>TEC	>PEC	EM (*n* = 44)	>TEC	>PEC
Ni	41 (93.2)	17 (38.6)	Ni	36 (81.2)	3 (6.8)
Cu	43 (97.7)	2 (14.3)	Cu	31 (70.5)	2 (4.5)
Zn	41 (93.2)	2 (14.3)	Zn	30 (68.2)	6 (13.6)
Pb	40 (90.9)	23 (52.3)	Pb	35 (79.5)	11 (25)
Hg (*n* = 35)	21 (60)	1 (2.9)	Hg (*n* = 40)	10 (25.6)	1 (2.6)
SW (*n* = 12)	>TEC	>PEC	LON (*n* = 5)	>TEC	>PEC
Ni	8 (66.7)	2 (16.7)	Ni	3 (60)	0
Cu	6 (50)	1 (8.3)	Cu	4 (80)	1 (20)
Zn	6 (50)	1 (8.3)	Zn	4 (80)	0
Pb	9 (75)	2 (16.7)	Pb	5 (100)	4 (80)
Hg (*n* = 10)	1 (10)	0	Hg (*n* = 4)	3 (75)	0
YH (*n* = 14)	>TEC	>PEC	EE (*n* = 67)	>TEC	>PEC
Ni	11 (78.5)	4 (28.6)	Ni	23 (34.3)	2 (3)
Cu	10 (71.4)	2 (14.3)	Cu	24 (35.8)	0
Zn	8 (57.1)	2 (14.3)	Zn	23 (34.3)	0
Pb	10 (71.4)	6 (42.9)	Pb	44 (65.7)	2 (3)
Hg (*n* = 13)	1 (7.7)	0	Hg (*n* = 45)	7 (15.6)	0
SE (*n* = 44)	>TEC	>PEC	England (*n* = 291)	>TEC	>PEC
Ni	16 (36.4)	1 (2.3)	Ni	178 (61.2)	39 (13.4)
Cu	18 (40.9)	3 (6.8)	Cu	181 (62.2)	33 (11.3)
Zn	20 (45.4)	4 (9.1)	Zn	168 (57.7)	39 (13.4)
Pb	25 (56.8)	3 (6.8)	Pb	218 (74.9)	68 (23.4)
Hg (*n* = 36)	5 (13.9)	0	Hg (*n* = 236)	70 (29.8)	6 (2.5)

Figures in brackets are percentage of sites >PEC in region. See Table 4 for region name abbreviations

### OPAL Metals Survey: significant contamination

Many water bodies in England, sampled during the survey, have sediment metal concentrations that exceed PEC sediment quality guidelines (Table 5). The number of samples that are statistical outliers (>90th percentile) (Fig. 5 and Table 6) in the dataset indicates a number of water bodies where a significant risk of severe ecological effect exists due to individual metal concentrations but especially in combination (mPEC-Q scores = ≥4). Current land use of these outlier sites indicates that these concentrations are likely the result of historical contamination by industry and current waste sources. In the notes provided by some of the participants, contamination from recent/contemporary sources (Table 6) is strongly suggested. A cluster of ponds in Bury, North West England (Unnamed Pond, OPM 202,217,218 Kirklees Valley) had the highest levels in the entire survey: Hg (Sample OPM 218, 3283 ng g^−1^), Zn (OPM 202, 5358 μg^−1^) and Pb (OPM 217, 1255 μg g^−1^) (Table 6). A reason to why these Bury water bodies contain high sediment metal concentrations is suggested by the notes submitted with the sample:

‘There are 6 separate ponds within the interior walls of an old mill (the walls are now only a few feet high and there is no roof/cover). Not sure if ponds formed after the roof was removed or if they were used within the mill for some purpose originally. They were there 40 years ago, in the same state, when I used to roam round there as a boy!’ Sample OPM202

**Table 6:** Selection of sites with significant (>90th percentile) elevated metal concentrations

	Code	Conc.	Conc. >PEC-Q	Location	Notes
Hg (ng)	*218*	3283	**2.9**	Unnamed Pond, Kirklees Valley, Bury	(See main text) ‘Built as mitigation for bypass, next to scrap metal plant’
	*601* ^a^	2539	**3.4**	Saltley Pool, Birmingham	
	334	2420	**1.6**	Bennion Pool, Leicester	No notes
	*217* ^a^	1665	**3.4**	Unnamed Pond, Kirklees Valley, Bury	(See main text)
	137	1530	**1.4**	Sankey Valley Park, Warrington	‘Rubbish dumped in pond, bikes and prams’
	517	1120	**1.7**	Clayton Vale Visitor Centre Pond, Manchester	No notes
Ni	594	282	**1.4**	Grove Park, Birmingham	‘...gullet through silt/oil trap to pond from church road’
	*599*	266	**2.9**	Goscote, Birmingham	‘…the pond was created as part of a larger environmental landscaping project in connection with a drainage scheme managed by Severn Trent Water. It seems to maintain good water level purely by ground water run off alone’
Cu	*595*	1164	**4.0**	Handsworth, West Bromwich	‘Old estates pool …now a heavily shaded small fishery’
	291	1005	**2.1**	Marazion Marsh, Cornwall	‘Pool surrounded by reed beds… Old tin mines in surrounding hills’
	*599*	1005	**2.9**	Goscote, Birmingham	(See above)
	*601* ^a^	605	**3.4**	Saltley Pool, Birmingham	(See above)
Zn	202	5358	**3.6**	Old Courtalds Mill, Bury	(See main text)
	*218*	2659	**2.9**	Unnamed Pond, Kirklees Valley, Bury	(See main text)
	073	2646	**2.0**	Argyle Street Pond, Hebburn	No notes
	*601* ^a^	2041	**3.4**	Saltley Pool, Birmingham	(See above)
	*595*	1861	**4.0**	Handsworth, West Bromwich	(See above)
	616	1727	**1.9**	Daphne Pool, Birmingham	‘Fluctuating in depth and nearly drying up at times, receives run off from surrounding rough grassland, though sites within a dense urban area, some contaminated runoff is obvious from nearby garage’
	*217* ^a^	1598	**3.4**	Unnamed Pond, Kirklees Valley, Bury	(See main text)
Pb	*217* ^a^	1255	**3.4**	Unnamed Pond, Kirklees Valley, Bury	(See main text)
	249	1120	**2.3**	Chaddesen Wood Pond, Derby	No notes
	403	872	**2.4**	The Tarn, Ilkley	‘Constructed by the Victorians. Dogs regularly swim in it. Large released terrapin are reputedly in residence’

Code is given upon receipt of samples. Two-metal occurrence was presented in data in italics. Concentrations are in micrograms per gram dry weight, except Hg (ng g^−1^). >PEC-Q = mean >PEC quotient for the five metals (MacDonald et al. 2000). Note text extracts were from information submitted with sample by participant

^a^Three metal occurrence

High Cu found in Marazion Marsh, Cornwall (OPM 291, 1005 μg g^−1^, 2.1 mPEC-Q) and elevated Pb (OPM 403, 872 μg g^−1^, 2.4 mPEC-Q) in Ilkley, Yorkshire may be due to a legacy of metal mining and processing. The second highest Pb concentration measured (OPM 249, 1120 μg g^−1^, 2.3 mPEC-Q) represents a less obvious cause; the sample is from a small woodland pond in a nature reserve in Derby. Contamination of small urban bodies should be no surprise in England due to the nation’s long history of intense industrial activity. Contemporary lakes and ponds occur in catchments where water provided both power and a mechanism for waste disposal. Many lakes and ponds were created and used intentionally to receive waste, while others not directly connected to inflows would have received significant inputs from atmospheric deposition. Because of contaminated soils in England (especially, but not exclusive to urban areas), excavated water bodies provide a mechanism for re-mobilisation of legacy pollutants.

## Conclusions

Guided but unassisted volunteers provide an accessible and viable pathway for sampling and assisting a national-scale assessment of metal concentrations in standing water bodies. The methodology used for participants to engage and generate sample returns was effective but limited in terms of systematic coverage of national extent and spatial density. Greater density sampling and spatial coverage could have been achieved by more directed voluntary sampling and effort on promotion of the survey. Participant-directed location of water bodies compared with a spatially and statistically robust sampling methodology leads to a reduced ability of upscaling results above the local scale.

The calibration exercise conducted in 10 lakes to assess the limitation of using a single-point littoral sample indicates that spatial homogeneity between littoral and deep water sediments largely occurs in small lakes (<20 ha) with non-peat-dominated catchments. Increased size of lake and heterogeneity of catchment in both upland and lowland areas increases variance of littoral sample concentrations.

The prevalence of small water bodies sampled by public participants and our calibration results supports the validity of this national assessment of sediment metal concentrations in standing water bodies. Applying consensus-based sediment quality guidelines to our concentration data shows that single and multiple metal element concentrations are likely having an ecological effect at many lakes and ponds in England and not exclusive to urban areas. With no history of statutory requirements to systematically sample and analyse standing water body sediment metal concentrations, and no national-scale baseline to compare them with, these data provide unique information on an overlooked factor of aquatic ecological health.

## Electronic supplementary material

Online Resource 1 (PDF 74 kb)

Online Resource 2 (PDF 81 kb)

Online Resource 3 (PDF 2954 kb)

Online Resource 4 (PDF 10285 kb)

Online Resource 5 (PDF 270 kb)

Online Resource 6 (PDF 308 kb)
