# Supplementary material for: Using public participation to sample trace metals in lake surface sediments: the OPAL Metals Survey
Source: Environ Monit Assess. 2017 Apr 28;189(5):241. doi: 10.1007/s10661-017-5946-y (PMC5409918; doi:10.1007/s10661-017-5946-y)
Supplement: Supplementary file 1 — (PDF 74 kb) [file 10661_2017_5946_MOESM1_ESM.pdf]

Online Resource 1. Site descriptions of calibration lakes. See also Table 1. Hyperlinks for more information on the lakes from the UK Lakes Portal are given.

Blea Tarn is a small (7.4 ha) and relatively deep (12m) natural upland tarn in the centre of the Lake District. The catchment geology is mainly rhyolite, andesite and tuff of the Borrowdale Volcanic Group (Ordovician). An extensive peat deposit forms the eastern side of the lake. Land use is upland rough grazing. More information on Blea Tarn.

Bonningtons Lake is a small (2.8 ha) lake in rural Hertfordshire, SE England. The catchment is formed by Palaeogene clays, sands and silts of the Lambeth Group and Thanet Formation and Quaternary glaciofluvial deposits. The lake is man-made (c. 19th century) and sits within a historic parkland estate that is now cultivated and also used for forestry. More information on Bonningtons Lake.

Burnmoor Tarn is a natural upland (253m asl) lake in the west of the Lake District, NW England, at the foot of Scafell Pike. The lake is underlain by rocks of Palaeozoic igneous origin; andesitic lavas of the Borrowdale Volcanic Group and in its eastern half by a granitic intrusion. Till and peat deposits cover the slopes surrounding the lake. Catchment land use is upland rough grazing. More information on Burnmoor Tarn

Compton Verney Lake is an ornamental lake situated in rural Warwickshire, central England. The lake is another construction by the 18th century landscaper Lancelot 'Capability' Brown. The catchment consists of Triassic and Jurassic sedimentary rocks with glacial till on higher ground. The lake itself overlies a narrow band of Penarth group mudstone. Catchment land-use is a mixture of intensive cultivation, parkland and woodland. More information on Compton Verney Lake.

Coombe Pool is a large (30.6 ha) ornamental lake situated in Coombe Abbey Country Park, on the outskirts of Coventry, central England. The 'serpentine' lake was constructed in the 18th century by damming a small stream during landscaping of estate grounds by Lancelot 'Capability' Brown. The area is underlain by Triassic deposits of the Mercia Mudstone Group. Adjacent to Coombe Pool are river terrace deposits and till. The catchment is a mixture of intensive cultivation, parkland and woodland. More information on Coombe Pool.

Hydelane Lake/Reservoir is situated about 2 km north east of Buckingham in southern England. The catchment comprises of Jurassic limestones and mudstones with a complex cover of Quaternary alluvial and glacial deposits. The lake was used for gravel extraction until the mid-20th century but it was originally constructed as a reservoir for the Buckingham Canal. Land use in the catchment consists of lowland mixed arable. More information on Hydelane Reservoir

Loweswater is a large natural lake (60 ha) in the north west of the Lake District. Catchment geology is Ordovician sedimentary rocks, with the lake mostly situated upon greywackes of the Loweswater Formation (Skiddaw Group). The lake sits in an alluvium filled glacial valley flanked by alluvial fan deposits. The catchment consists of lowland mixed arable and upland grazing. More information on Loweswater.

Preston's Lake is a man-made lake on the outskirts of Halstead, Essex, SE England. Catchment solid geology is London Clay (Palaeogene) overlain by a complex of alluvial and glacial drift. The lake was constructed in the 1990s as a fishing lake. Land use in the catchment is intensive farming and rural settlements. The UK Lakes Portal does not record Preston's Lake.

Scampston Park Lake (4.9 ha) sits in a lowland (32 m asl) valley in Yorkshire, NE England. It is an ornamental 'serpentine' lake constructed during landscaping in the 18th century by Lancelot 'Capability' Brown. The catchment is situated over Jurassic mudstones of the Ampthill and Kimmeridge Clay Formations, which is covered by sand and gravel deposits. Streams entering the lake also derive from Cretaceous chalk springs. Land use is primarily mixed arable agriculture. More information on Scampston Park Lake.

Stickle Tarn is a small (7.4 ha) upland tarn in the Lake District. The catchment consists of Ordovician volcanics, specifically tuff, and hummocky glacial deposits, talus and peat soils. Catchment land use is upland rough grazing. The lake has a dam constructed in the early 19th century to maintain levels for water supply. More information on Stickle Tarn.
