# Supplementary material for: Using public participation to sample trace metals in lake surface sediments: the OPAL Metals Survey
Source: Environ Monit Assess. 2017 Apr 28;189(5):241. doi: 10.1007/s10661-017-5946-y (PMC5409918; doi:10.1007/s10661-017-5946-y)
Supplement: Supplementary file 2 — (PDF 81 kb) [file 10661_2017_5946_MOESM2_ESM.pdf]

Online Resource 2: Results from inter-laboratory tests between British Geological Survey and UCL Geography.

(a) Inter-laboratory element concentrations and %difference (%D) of stream and soil samples BGS G-Base archive measured (b) Inter-laboratory element concentrations and %difference (%D) of reference sediment values (CanMet LKSD2) and %D (c) Comparison of reported and measured values of LKSD-2 reference sediment during runs of OPAL Metals Survey samples

**(a)**		**Ni (μg g^{1})**			**Cu (μg g^{1})**			**Zn (μg g^{1})**			**Pb (μg g^{1})**		
**Site**	**Code**	**UCL**	**BGS**	**%D**	**UCL**	**BGS**	**%D**	**UCL**	**BGS**	**%D**	**UCL**	**BGS**	**%D**
Blea	C 322772	25.6	27.1	5.9	10.6	10.8	2.5	387.5	387.7	0.06	32.8	36.3	10.4
Bonnington's	S 449297	31.3	30.2	3.5	16.9	20.5	19.2	80.6	87	7.6	37	36.8	0.5
Compton	S 430924	29.9	20.1	39.2	18.3	14.8	21.1	71.8	62.2	14.3	29.1	29.2	0.3
Coombe	C 426891	38.4	39.9	3.8	36	35	2.8	168.7	166.9	1.0	49	47.3	3.5
Coombe	S 609190	37.9	29	26.6	32.7	28	15.4	136.8	103	28.1	83.3	66	23.1
Preston's	S 450740	49.9	39.4	23.5	24.4	20.3	18.3	93.2	76	20.3	35.3	31	12.9
Scampston	S 305204	27.6	24	13.9	21.7	14	43.1	76.8	82	6.5	35.6	31	13.8
**(b)**													
**LKSD2 Reference Sed.**		**UCL**	**BGS**	**%D**	**UCL**	**BGS**	**%D**	**UCL**	**BGS**	**%D**	**UCL**	**BGS**	**%D**
		27.8	26	6.9	35.1	37	5.2	201.9	209	3.4	44.4	44	0.9

**(c)**	**Ni (μg g^{1})**			**Cu (μg g^{1})**			**Zn (μg g^{1})**			**Pb (μg g^{1})**		
	**UCL**	**LKSD2**	**%D**	**UCL**	**LKSD2**	**%D**	**UCL**	**LKSD2**	**%D**	**UCL**	**LKSD2**	**%D**
OPAL Water Survey sample runs (n=28); mean [sd]	27.6 [3.1]	26 [4]	6.0	35.2 [3.1]	37 [4]	5.0	195.5 [9.8]	209 [18]	6.7	44.4 [2.4]	44 [4]	0.9
