# Supplementary material for: Using public participation to sample trace metals in lake surface sediments: the OPAL Metals Survey
Source: Environ Monit Assess. 2017 Apr 28;189(5):241. doi: 10.1007/s10661-017-5946-y (PMC5409918; doi:10.1007/s10661-017-5946-y)

Online Resource 3: (a) Blea Tarn: Sample Location map and PCA of LOI, Hg, Ni, Zn, Cu, Pb. Unit standardised and log-normalised. See Table 1 for lake location and details. Basemap © Crown Copyright and Database Right 2016. Ordnance Survey (Digimap Licence)

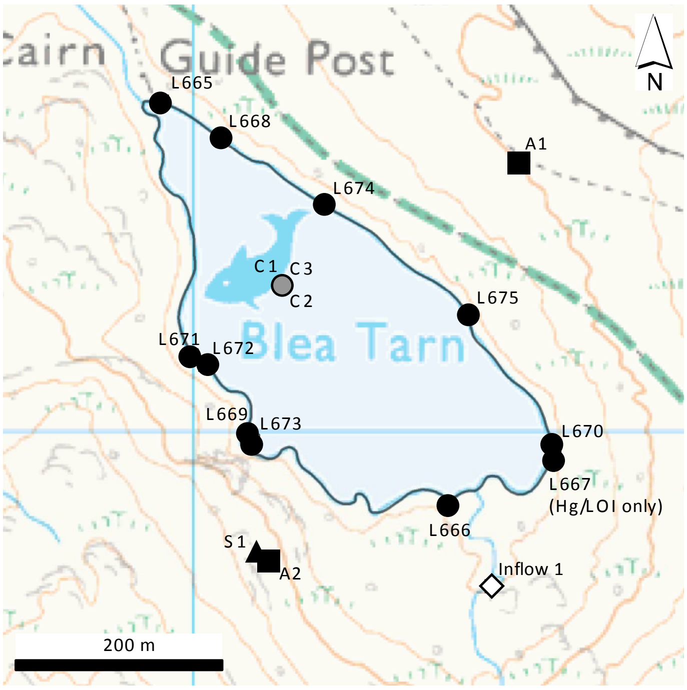

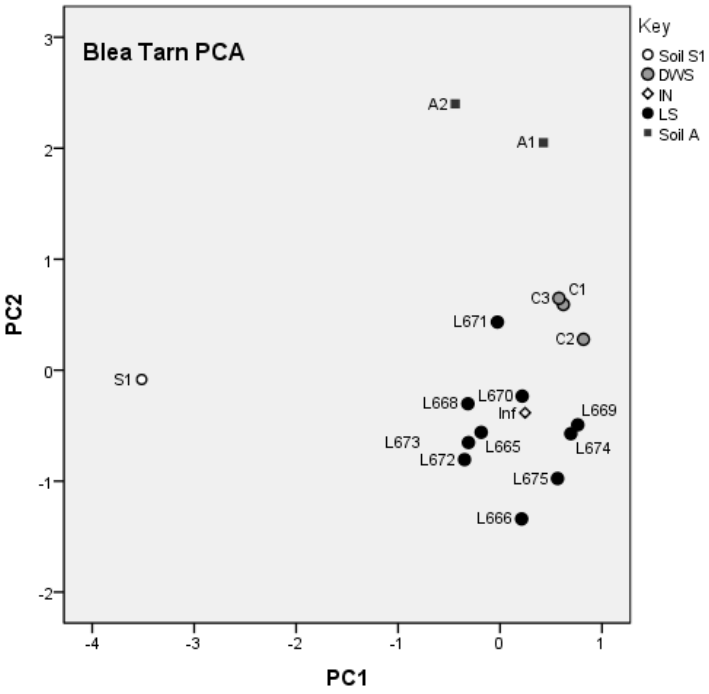

Online Resource 3: (b) Bonningtons Lake: Sample Location map and PCA of LOI, Hg, Ni, Zn, Cu, Pb. Unit standardised and log-normalised. See Table 1 for lake location and details. Basemap © Crown Copyright and Database Right 2016. Ordnance Survey (Digimap Licence)

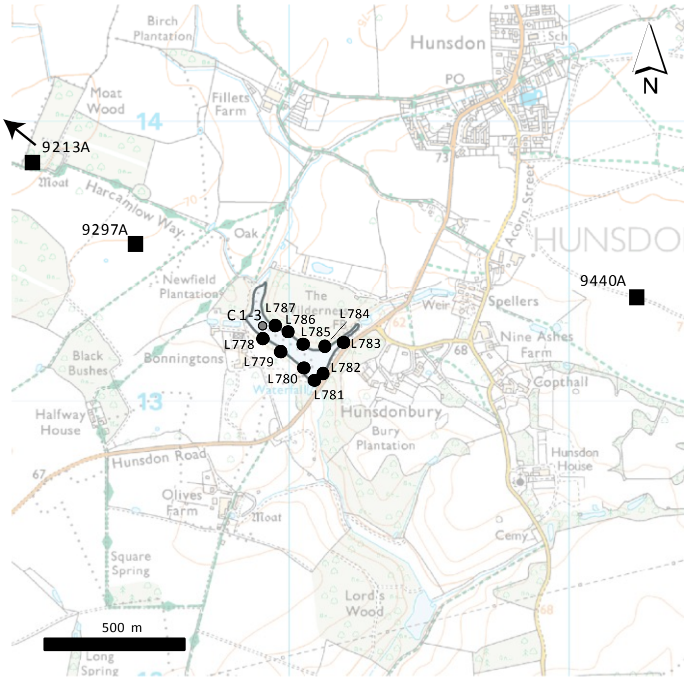

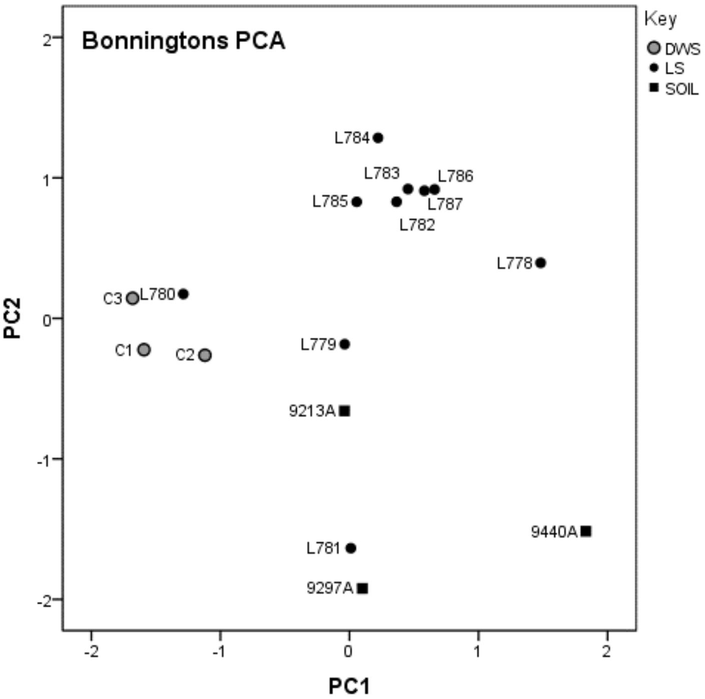

Online Resource 3: (c) Burnmoor Tarn: Sample Location map and PCA of LOI, Hg, Ni, Zn, Cu, Pb. Unit standardised and log-normalised. See Table 1 for lake location and details. Basemap © Crown Copyright and Database Right 2016. Ordnance Survey (Digimap Licence)

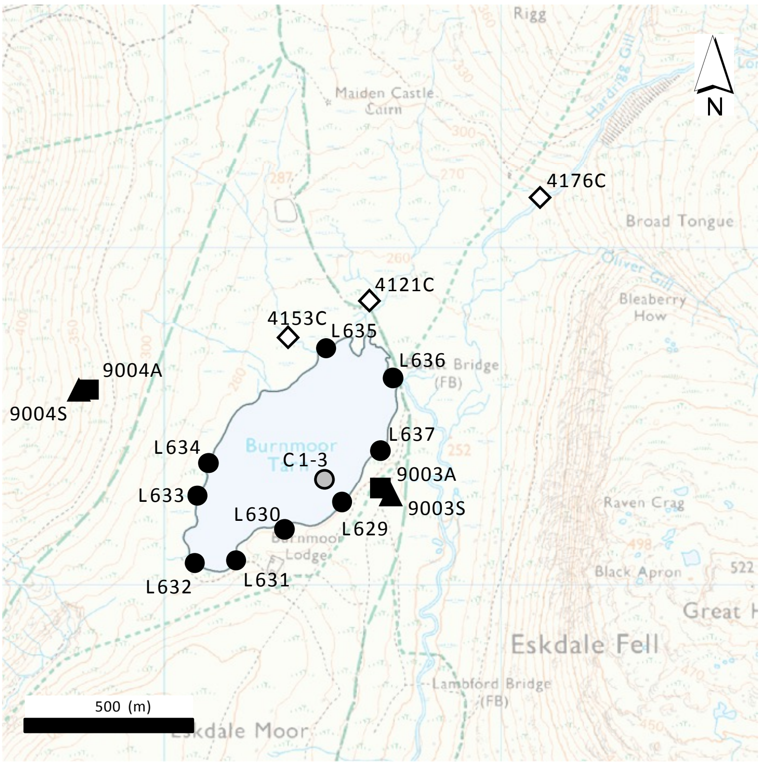

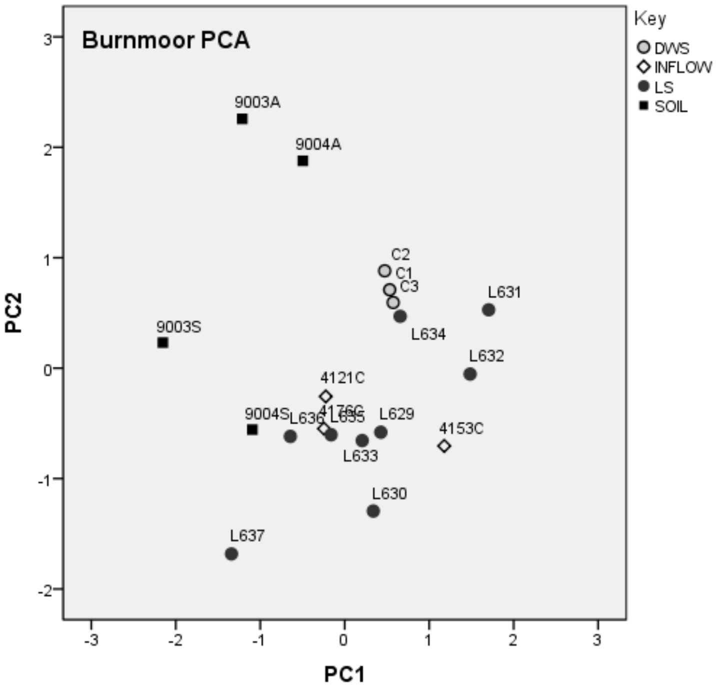

Online Resource 3: (d) Compton Verney: Sample Location map and PCA of LOI, Hg, Ni, Zn, Cu, Pb. Unit standardised and log-normalised. See Table 1 for lake location and details. Basemap © Crown Copyright and Database Right 2016. Ordnance Survey (Digimap Licence)

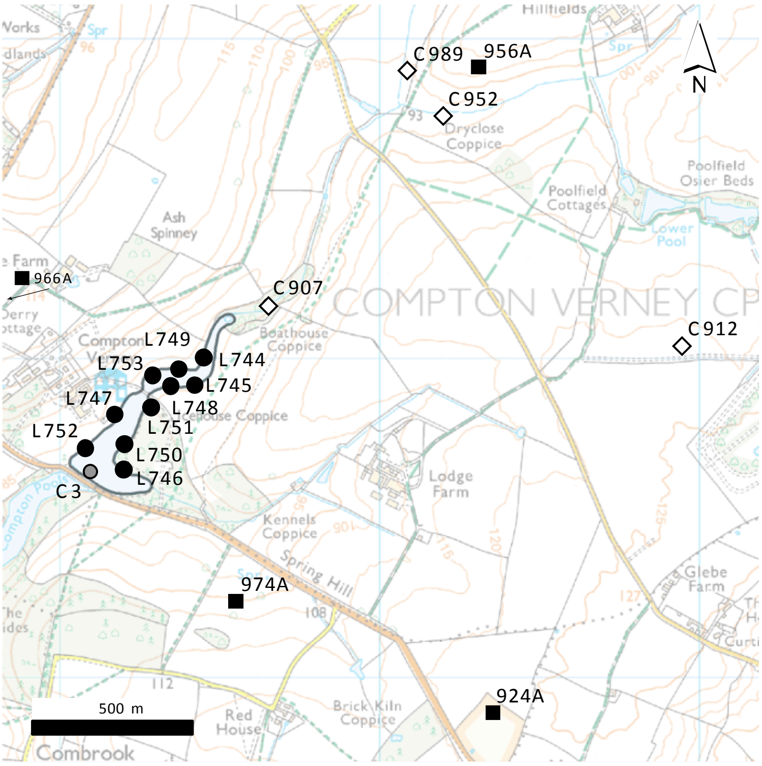

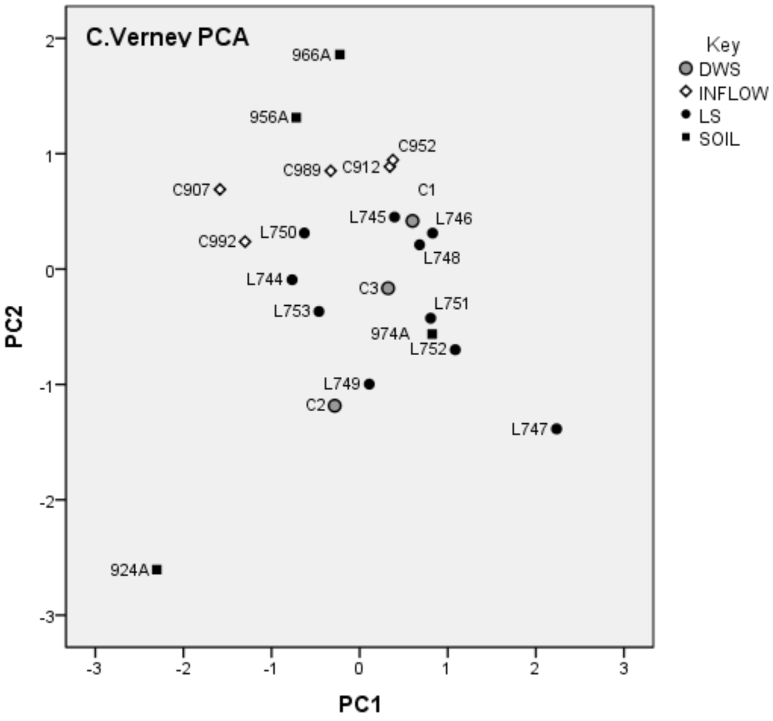

Online Resource 3: (e) Coombe Pool: Sample Location map and PCA of LOI, Hg, Ni, Zn, Cu, Pb. Unit standardised and log-normalised. See Table 1 for lake location and details. Basemap © Crown Copyright and Database Right 2016. Ordnance Survey (Digimap Licence)

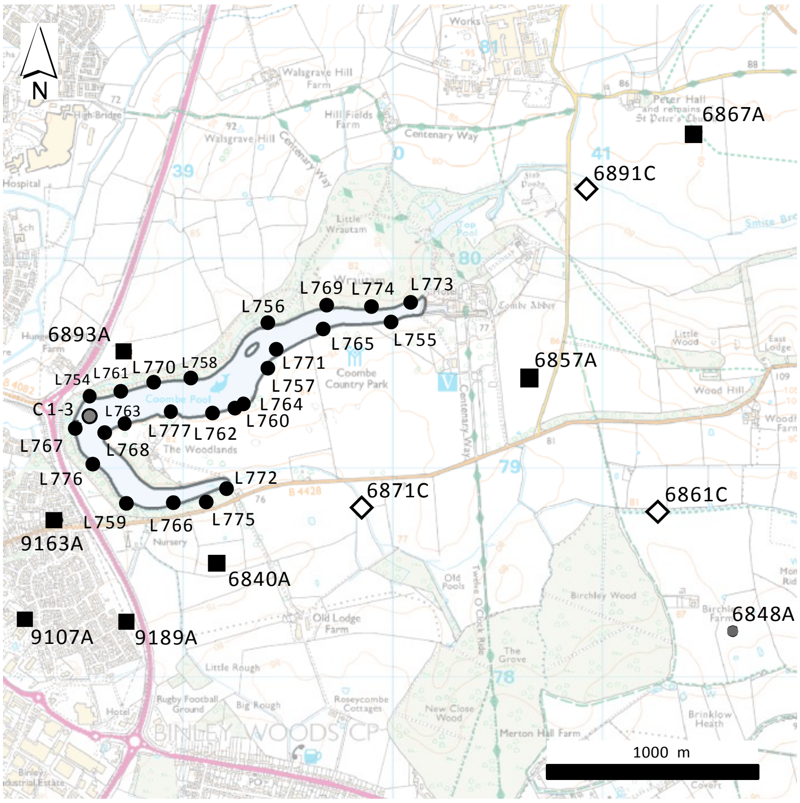

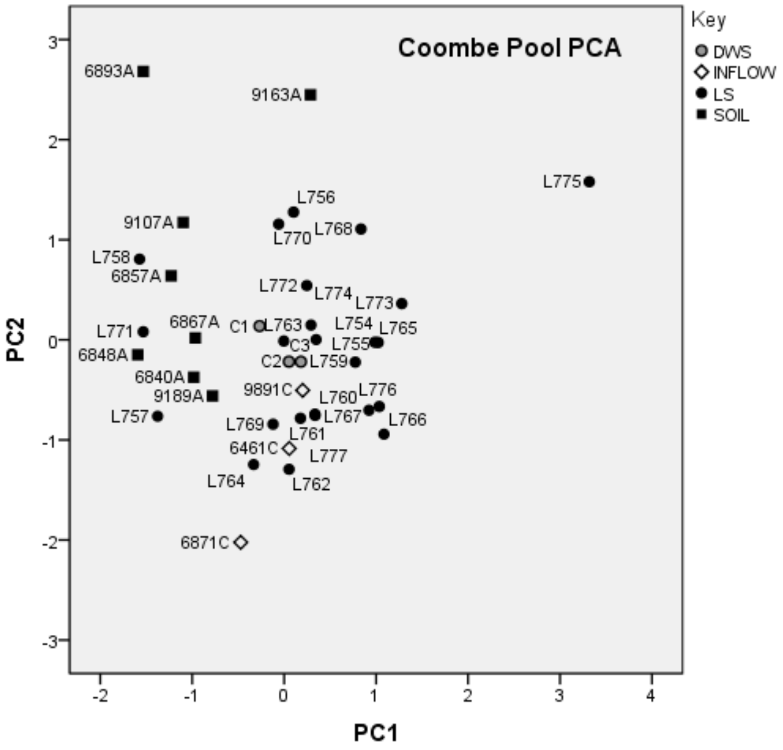

Online Resource 3: (f) Hydelane Reservoir: Sample Location map and PCA of LOI, Hg, Ni, Zn, Cu, Pb. Unit standardised and log-normalised. See Table 1 for lake location and details. Basemap © Crown Copyright and Database Right 2016. Ordnance Survey (Digimap Licence)

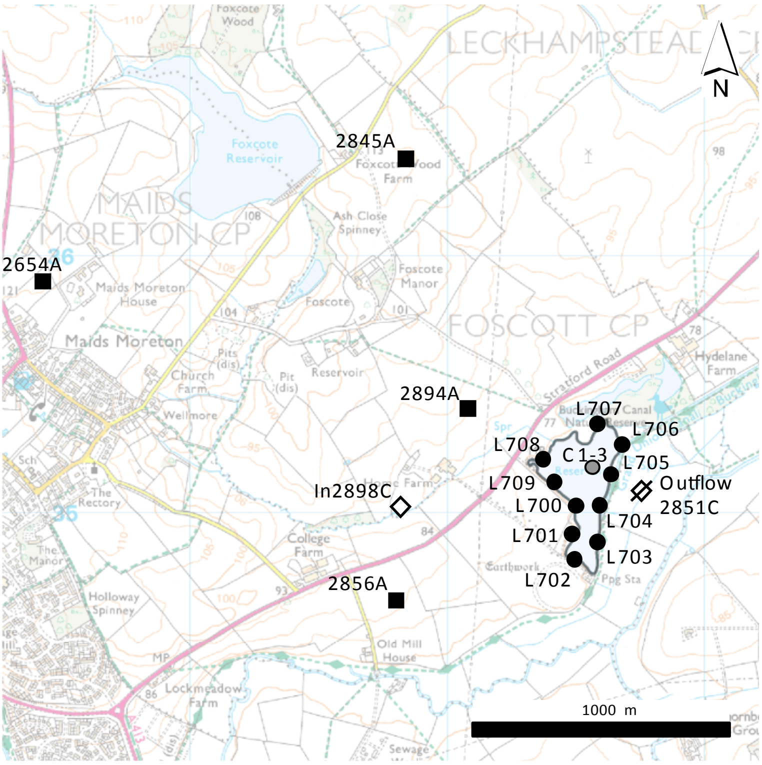

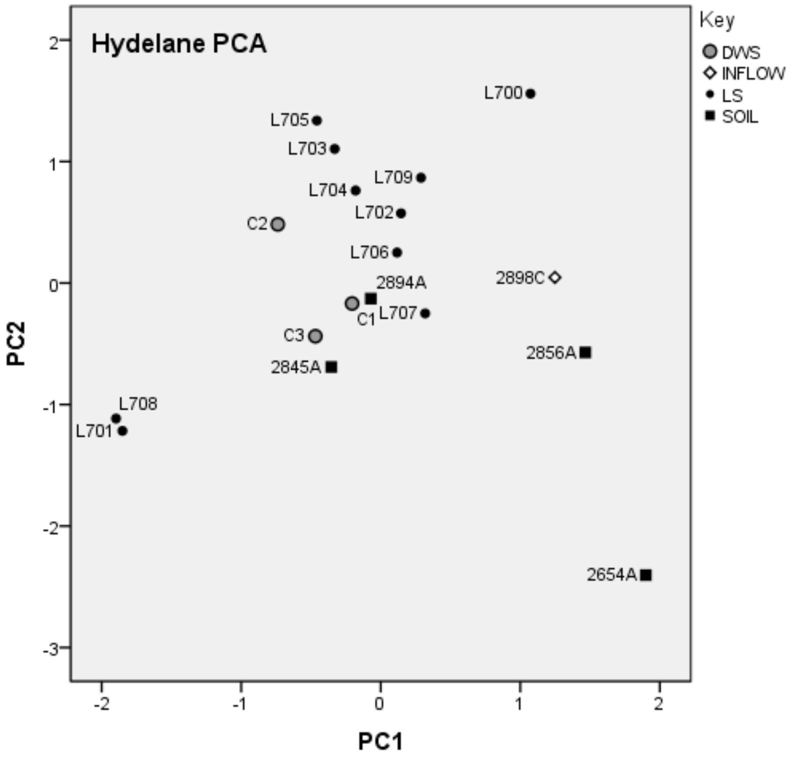

Online Resource 3: (g) Loweswater: Sample Location map and PCA of LOI, Hg, Ni, Zn, Cu, Pb. Unit standardised and log-normalised. See Table 1 for lake location and details. Basemap © Crown Copyright and Database Right 2016. Ordnance Survey (Digimap Licence)

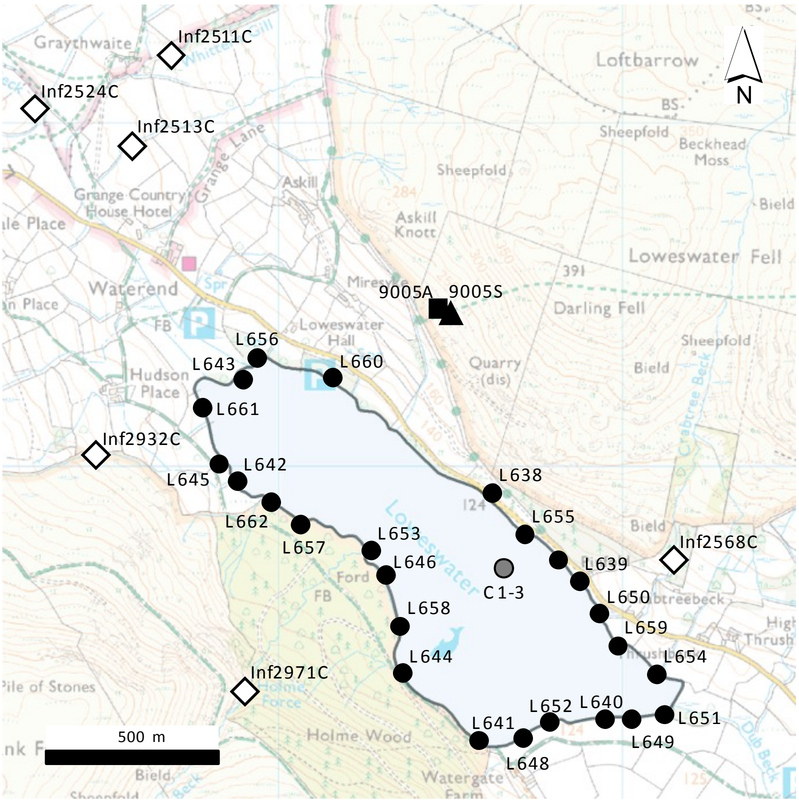

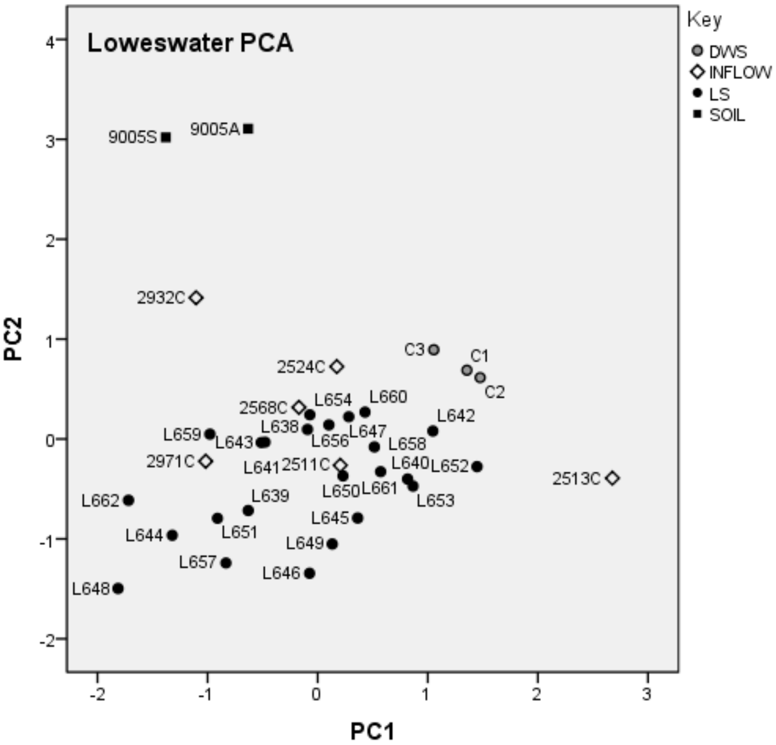

Online Resource 3: (h) Preston's Lake: Sample Location map and PCA of LOI, Hg, Ni, Zn, Cu, Pb. Unit standardised and log-normalised. See Table 1 for lake location and details. Basemap © Crown Copyright and Database Right 2016. Ordnance Survey (Digimap Licence)

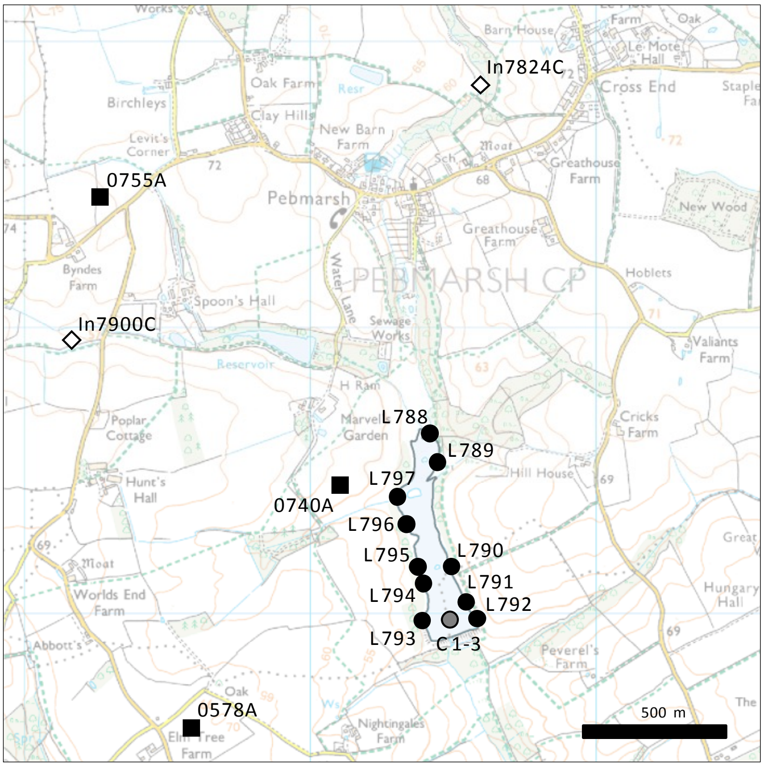

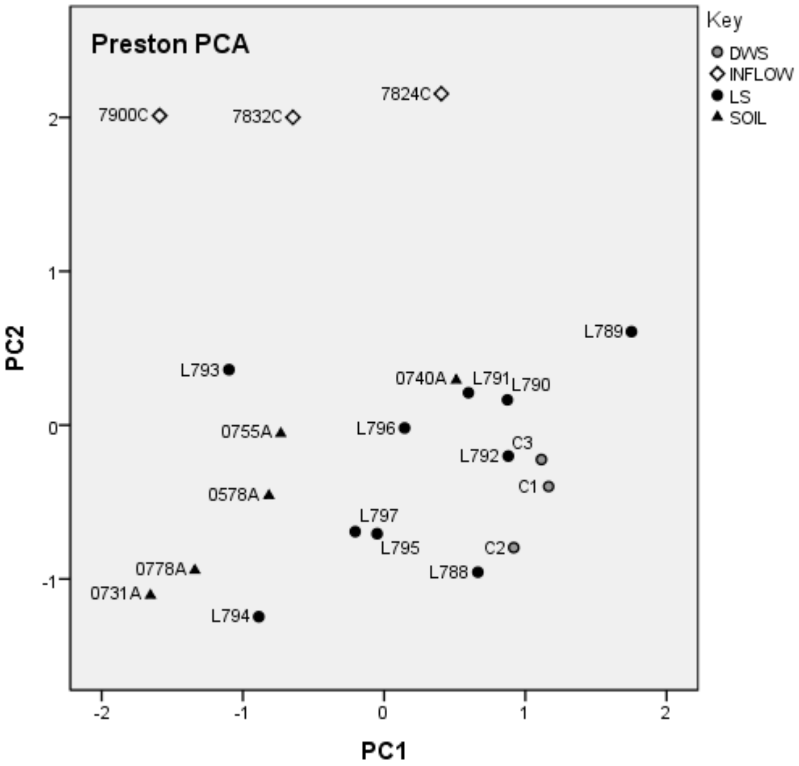

Online Resource 3: (i) Scampston Lake: Sample Location map and PCA of LOI, Hg, Ni, Zn, Cu, Pb. Unit standardised and log-normalised. See Table 1 for lake location and details. Basemap © Crown Copyright and Database Right 2016. Ordnance Survey (Digimap Licence)

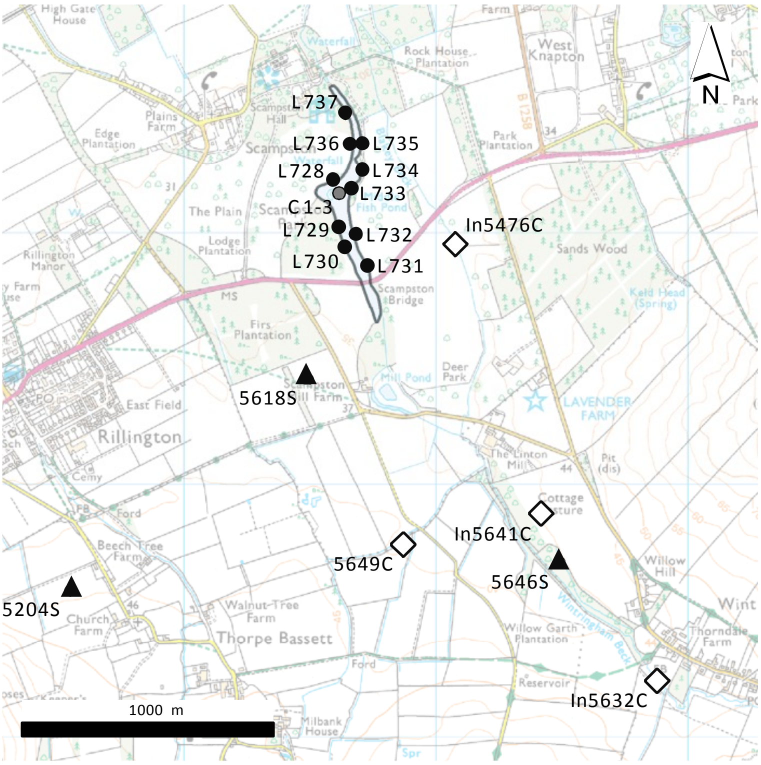

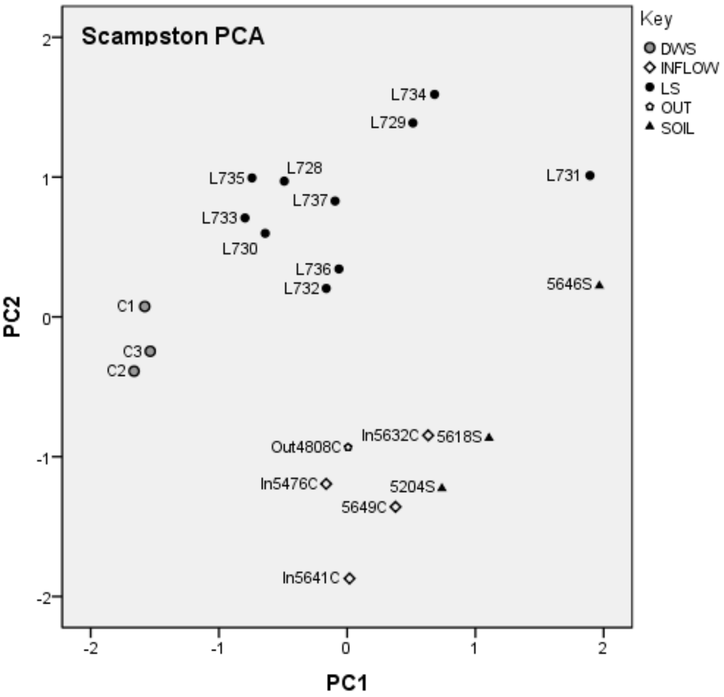

Online Resource 3: (j) Stickle Tarn: Sample Location map and PCA of LOI, Hg, Ni, Zn, Cu, Pb. Unit standardised and log-normalised. See Table 1 for lake location and details. Basemap © Crown Copyright and Database Right 2016. Ordnance Survey (Digimap Licence)

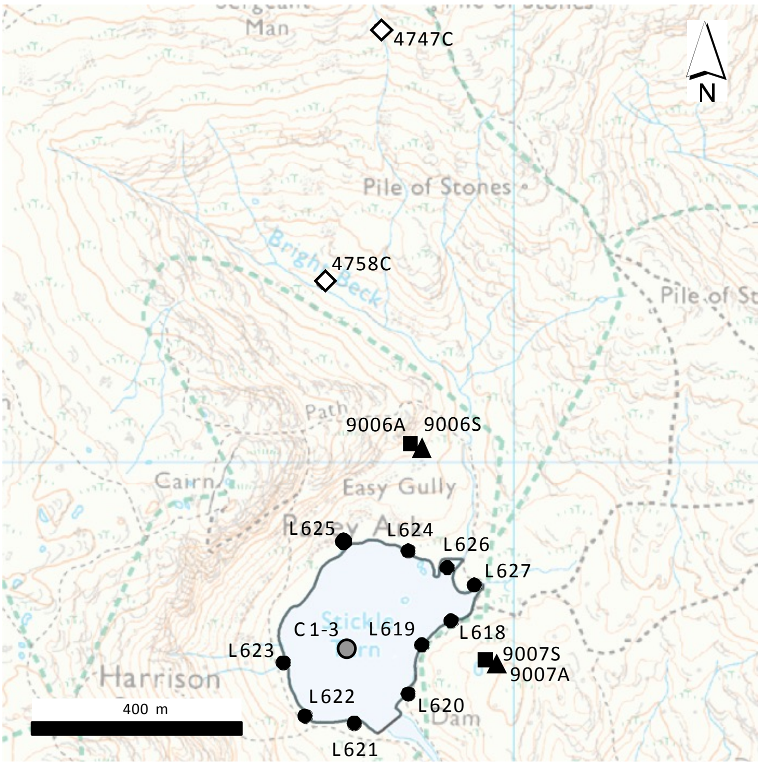

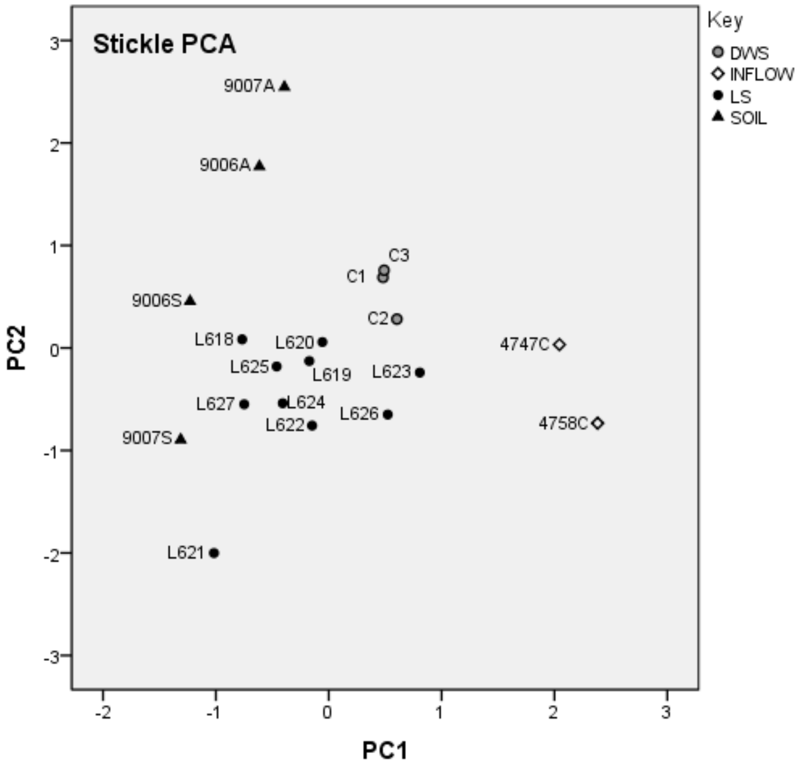

Supplement: Supplementary file 3 — (PDF 2954 kb) [file 10661_2017_5946_MOESM3_ESM.pdf]
