# Supplementary material for: Using public participation to sample trace metals in lake surface sediments: the OPAL Metals Survey
Source: Environ Monit Assess. 2017 Apr 28;189(5):241. doi: 10.1007/s10661-017-5946-y (PMC5409918; doi:10.1007/s10661-017-5946-y)

Online Resource 4: (a) Blea Tarn. See Table 1 for lake location and details. Classification of metal element concentrations by <TEC (green), >TEC (yellow) and >PEC (red). Basemap © Crown Copyright and Database Right 2016. Ordnance Survey (Digimap Licence)

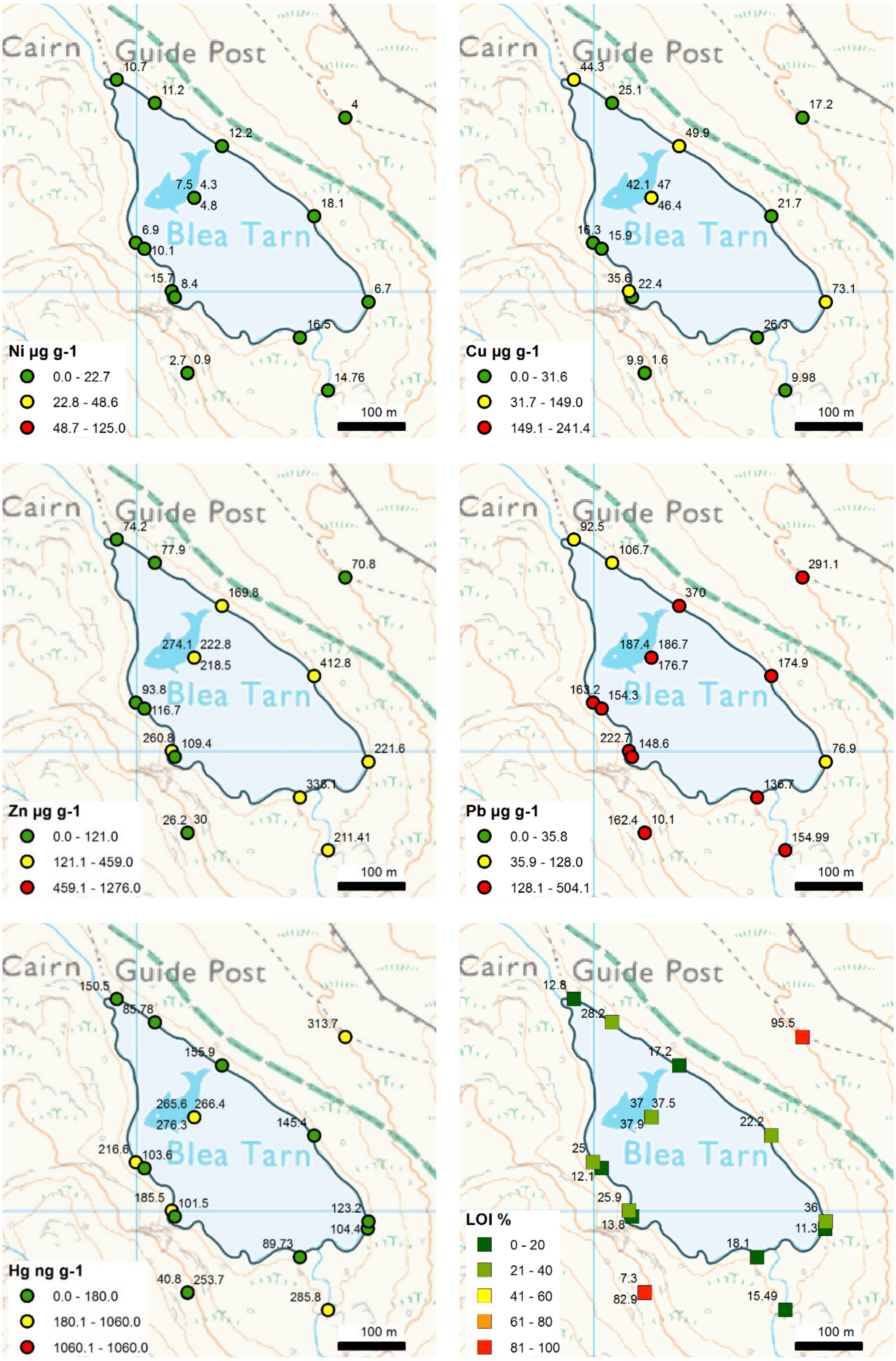

Online Resource 4: (b) Bonnington's Lake. See Table 1 for lake location and details. Classification of metal element concentrations by <TEC (green), >TEC (yellow) and >PEC (red). Basemap © Crown Copyright and Database Right 2016. Ordnance Survey (Digimap Licence)

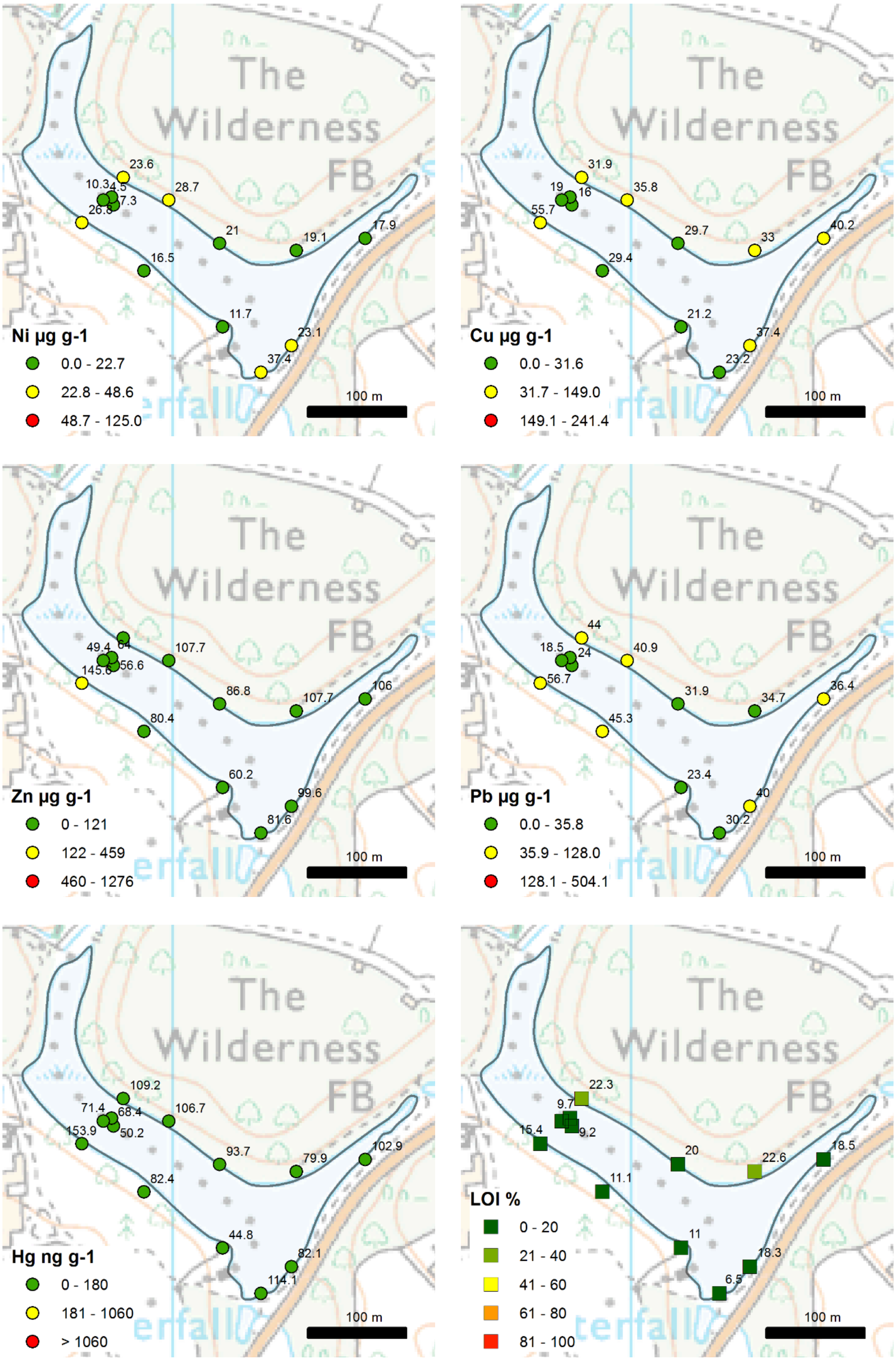

Online Resource 4: (c) Burnmoor Tarn. See Table 1 for lake location and details. Classification of metal element concentrations by <TEC (green), >TEC (yellow) and >PEC (red). Basemap © Crown Copyright and Database Right 2016. Ordnance Survey (Digimap Licence).

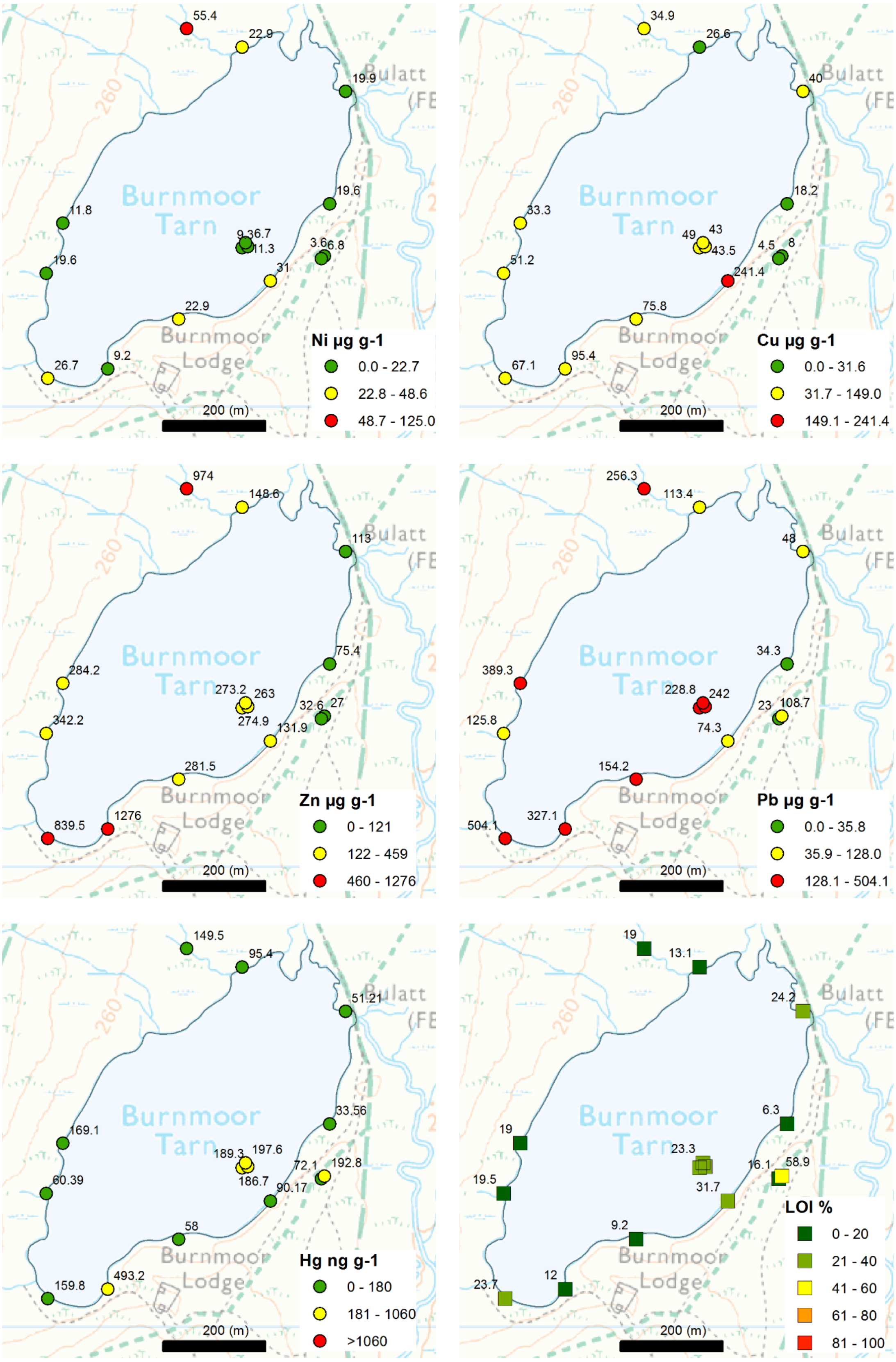

Online Resource 4: (d) Compton Verney. See Table 1 for lake location and details. Classification of metal element concentrations by <TEC (green), >TEC (yellow) and >PEC (red). Basemap © Crown Copyright and Database Right 2016. Ordnance Survey (Digimap Licence)

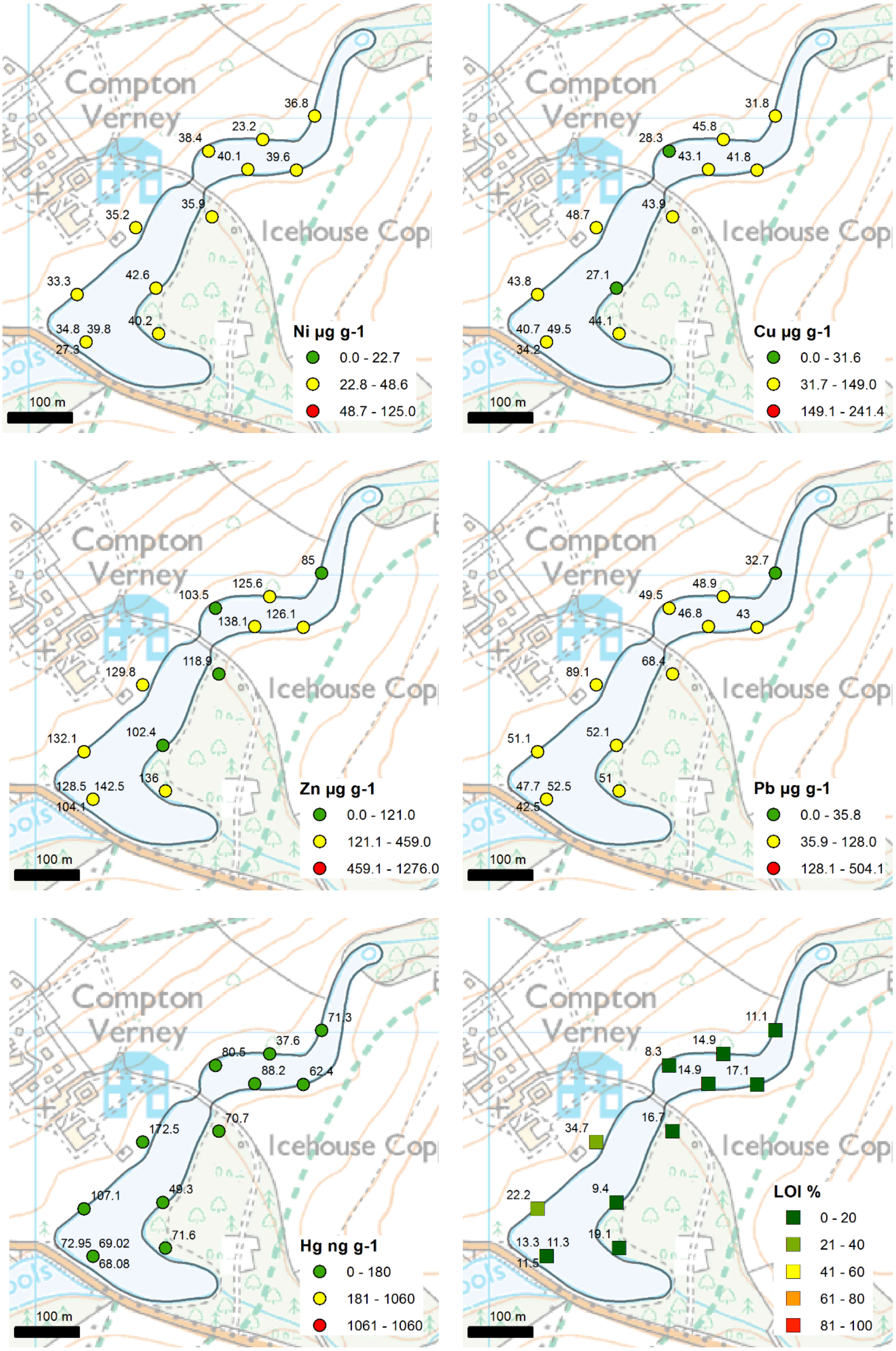

Online Resource 4: (e) Coombe Pool. See Table 1 for lake location and details. Classification of metal element concentrations by <TEC (green), >TEC (yellow) and >PEC (red). Basemap © Crown Copyright and Database Right 2016. Ordnance Survey (Digimap Licence)

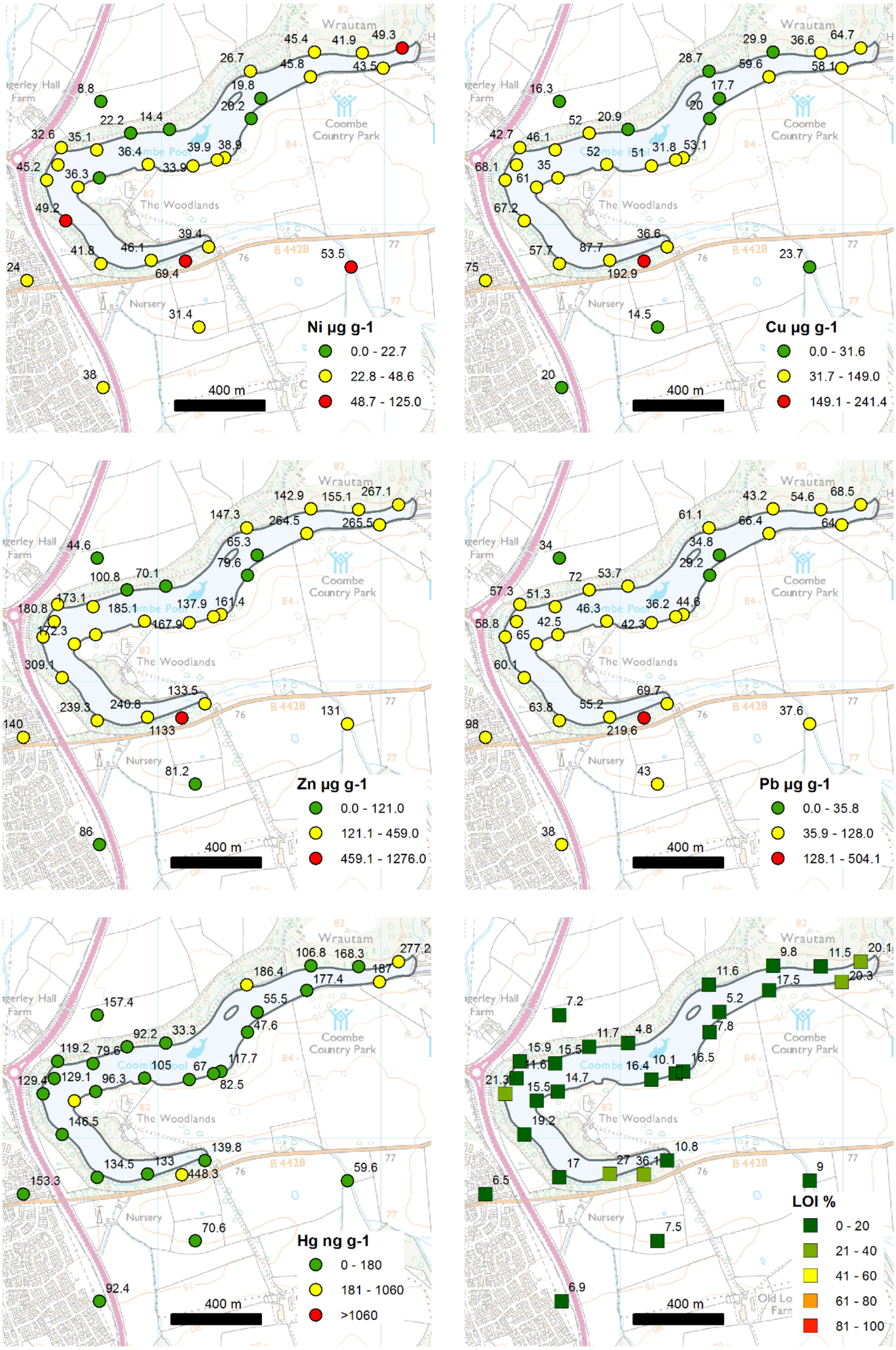

Online Resource 4: (f) Hydelane Reservoir. See Table 1 for lake location and details. Classification of metal element concentrations by <TEC (green), >TEC (yellow) and >PEC (red). Basemap © Crown Copyright and Database Right 2016. Ordnance Survey (Digimap Licence)

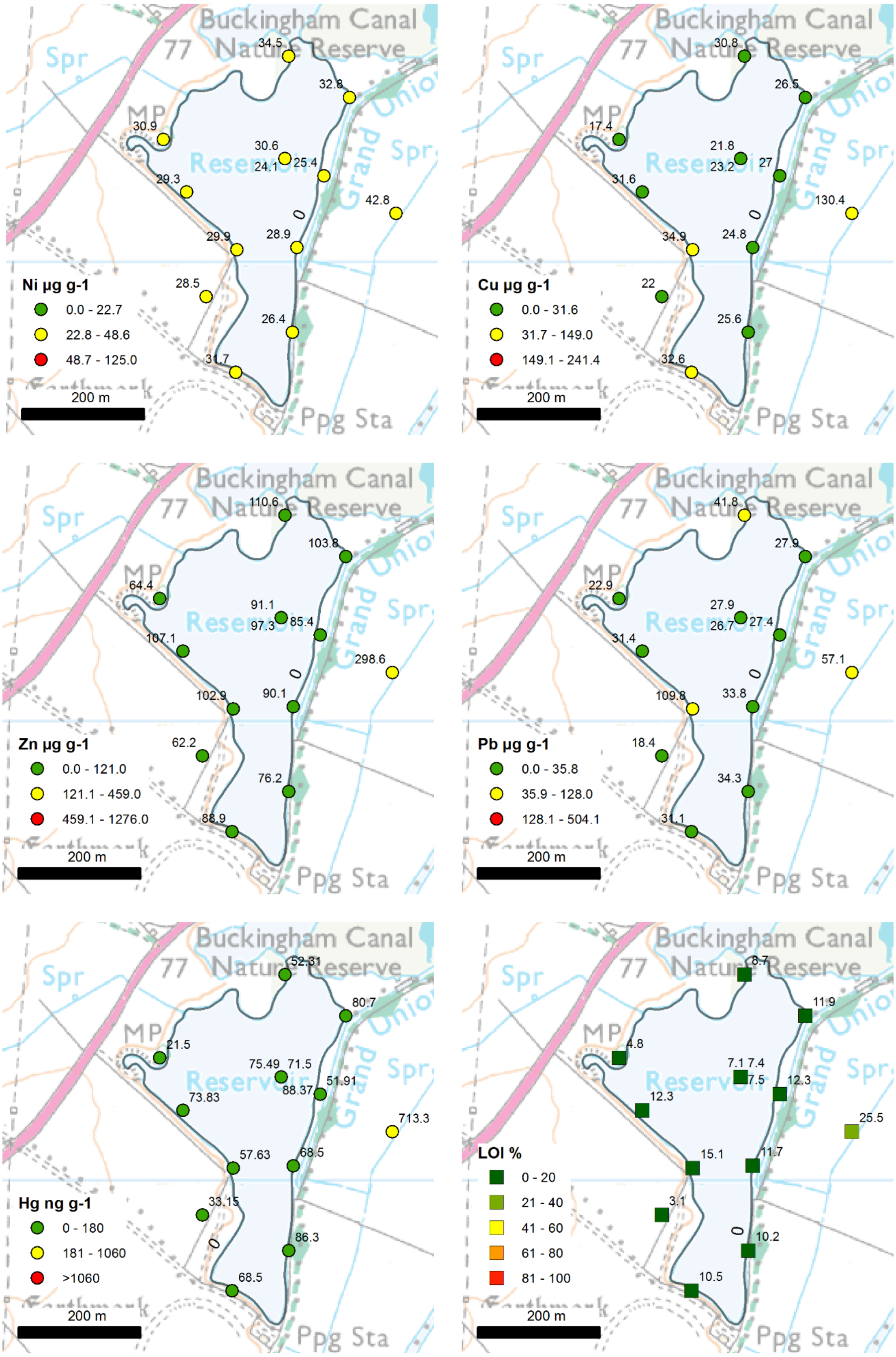

Online Resource 4: (g) Loweswater. See Table 1 for lake location and details. Classification of metal element concentrations by <TEC (green), >TEC (yellow) and >PEC (red). Basemap © Crown Copyright and Database Right 2016. Ordnance Survey (Digimap Licence)

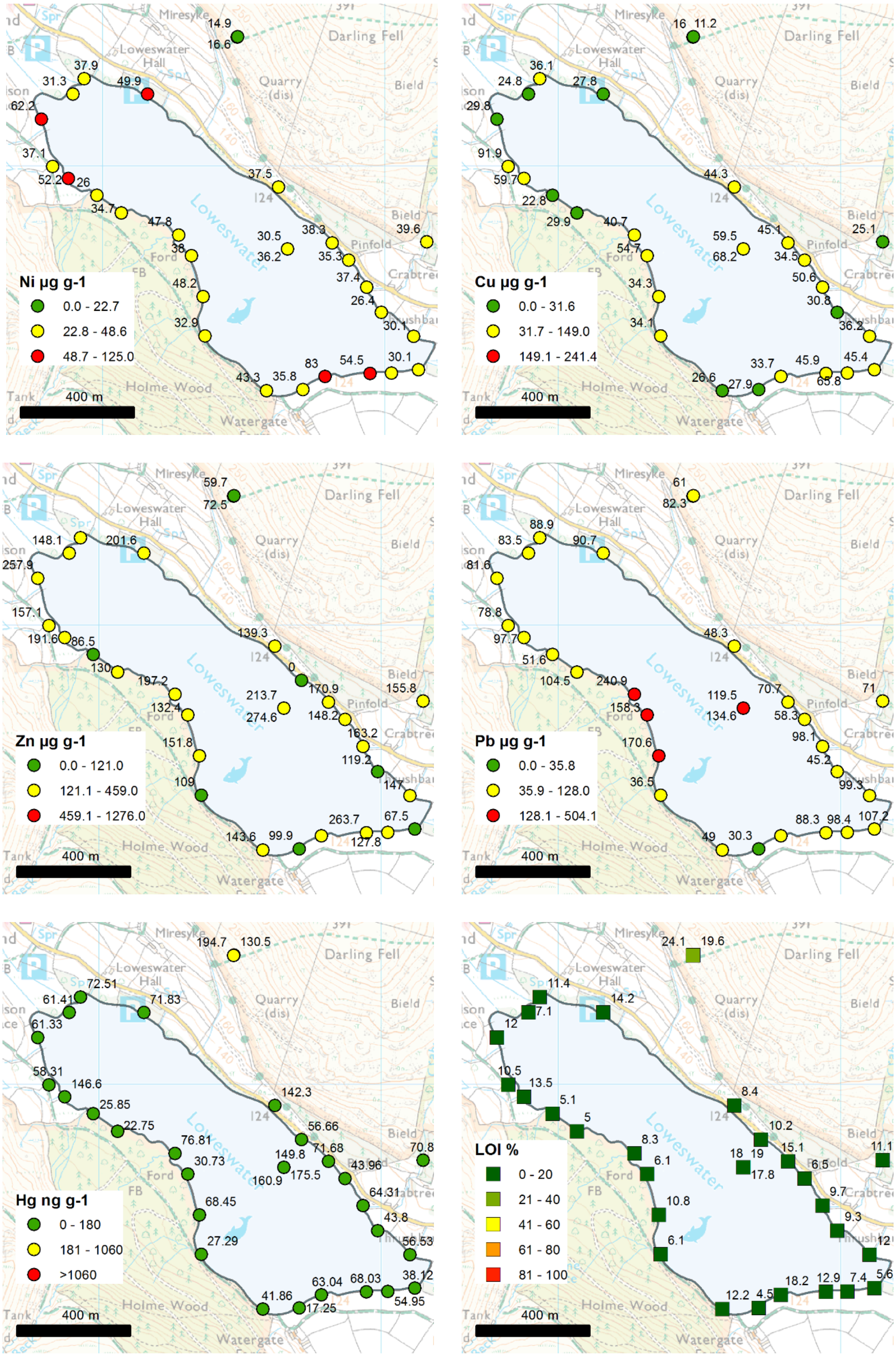

Online Resource 4: (h) Preston's Lake. See Table 1 for lake location and details. Classification of metal element concentrations by <TEC (green), >TEC (yellow) and >PEC (red). Basemap © Crown Copyright and Database Right 2016. Ordnance Survey (Digimap Licence)

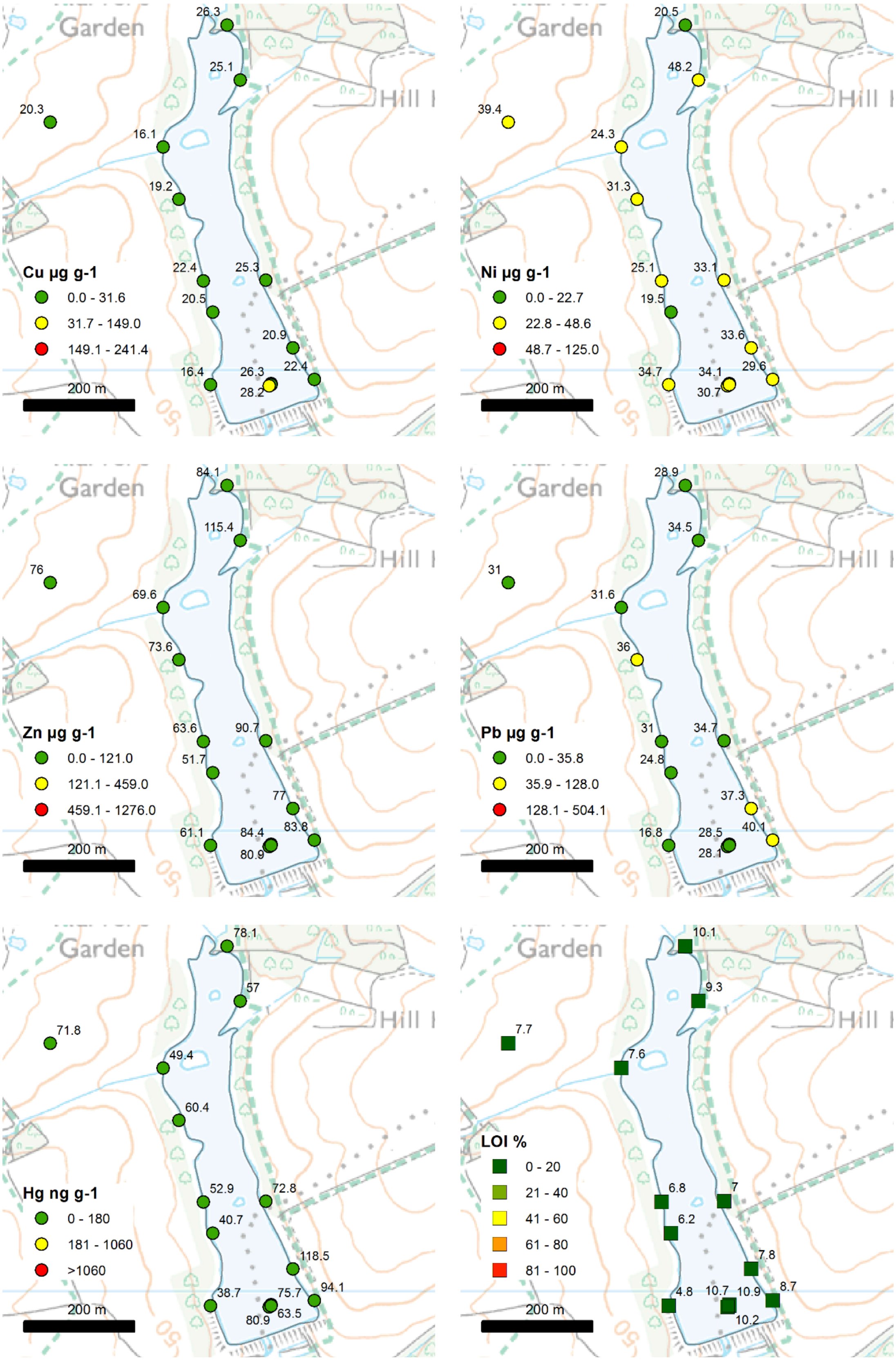

Online Resource 4: (i) Scampston Lake. See Table 1 for lake location and details. Classification of metal element concentrations by <TEC (green), >TEC (yellow) and >PEC (red). Basemap © Crown Copyright and Database Right 2016. Ordnance Survey (Digimap Licence)

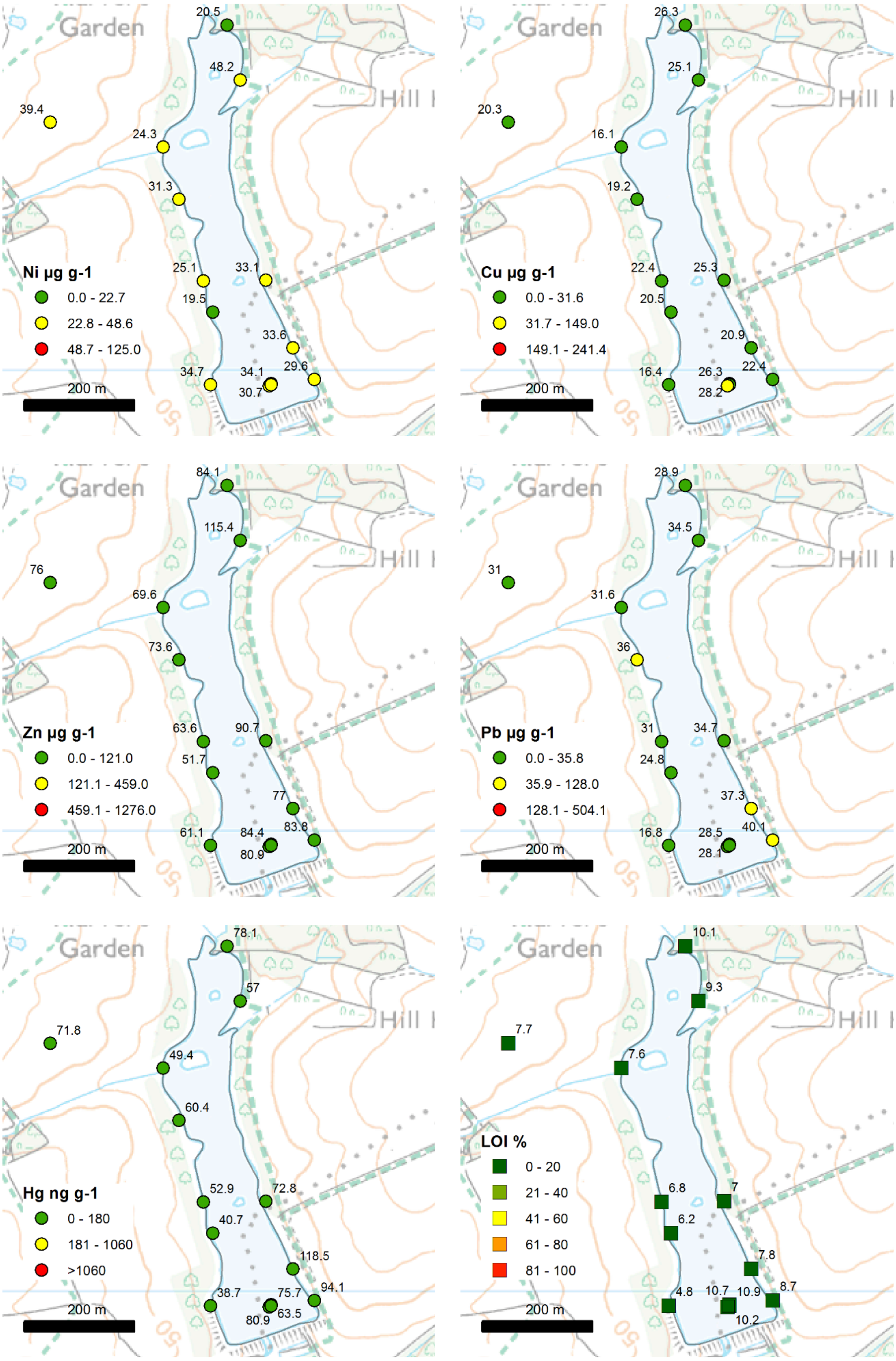

Online Resource 4: (j) Stickle Tarn. See Table 1 for lake location and details. Classification of metal element concentrations by <TEC (green), >TEC (yellow) and >PEC (red). Basemap © Crown Copyright and Database Right 2016. Ordnance Survey (Digimap Licence)

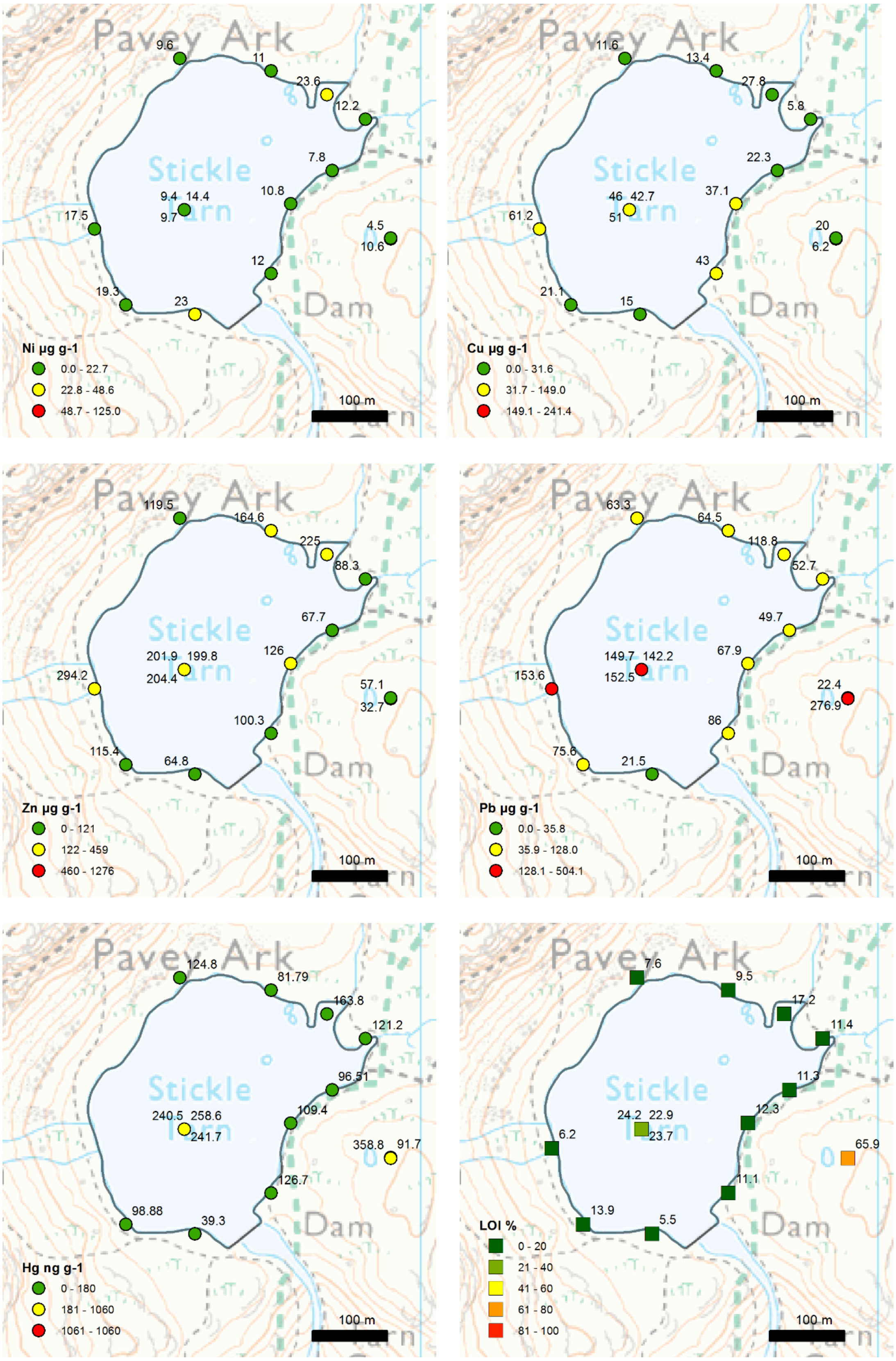

Supplement: Supplementary file 4 — (PDF 10285 kb) [file 10661_2017_5946_MOESM4_ESM.pdf]
