# Supplementary material for: Using public participation to sample trace metals in lake surface sediments: the OPAL Metals Survey
Source: Environ Monit Assess. 2017 Apr 28;189(5):241. doi: 10.1007/s10661-017-5946-y (PMC5409918; doi:10.1007/s10661-017-5946-y)

Online Resource 5: Percentage difference (%D) between individual littoral metal sample concentrations and mean (Hubers) of all littoral sediments around calibration lakes.

Burnmoor Tarn

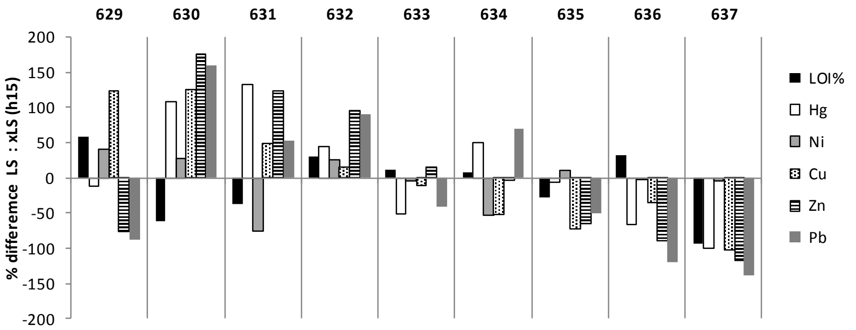

Stickle Tarn

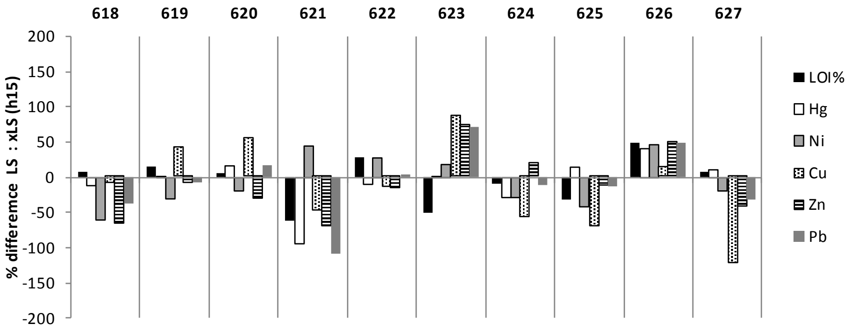

Blea Tarn

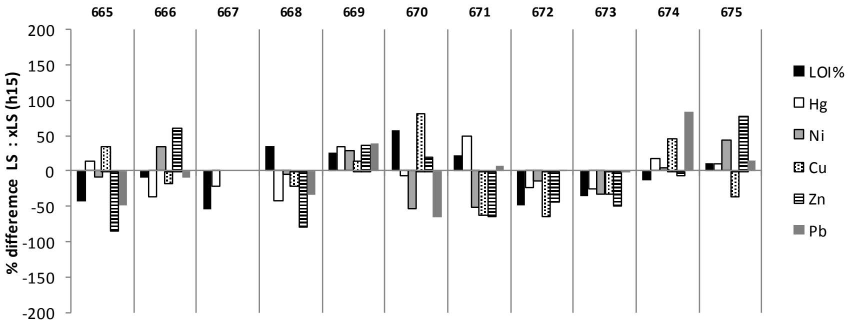

Hydelane

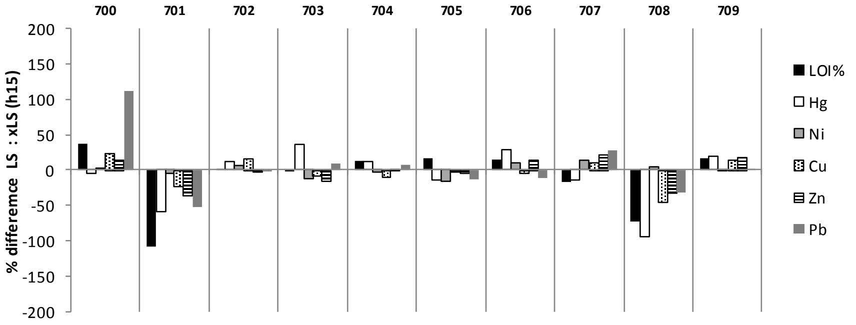

Online Resource 5: Percentage differences between littoral metal concentrations and mean (Hubers) of littoral sediments around calibration lakes.

Bonningtons

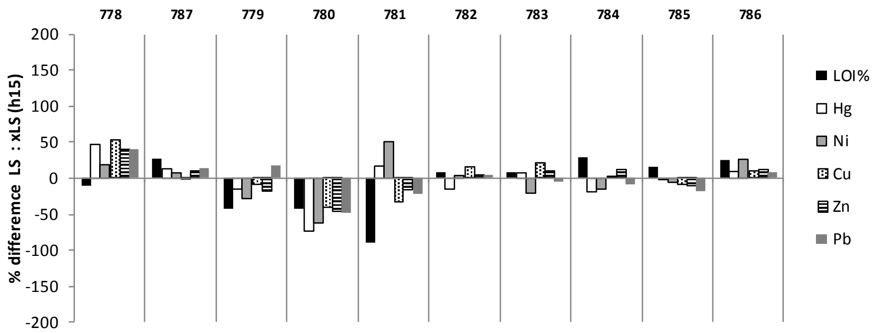

Prestons Lake

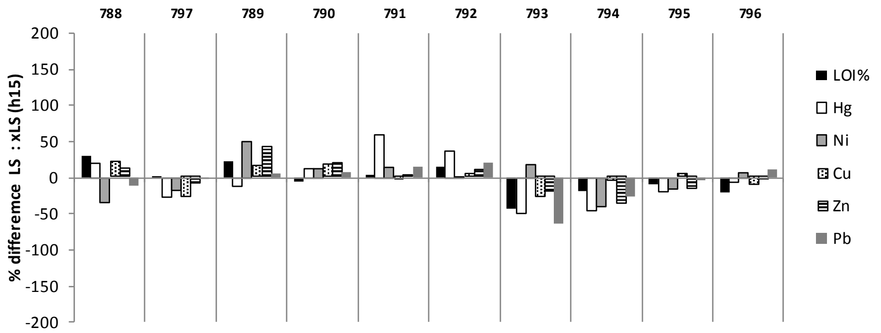

Compton Verney

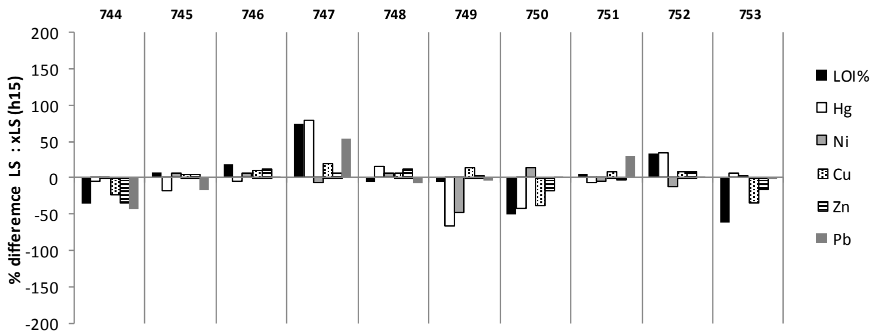

Scampston

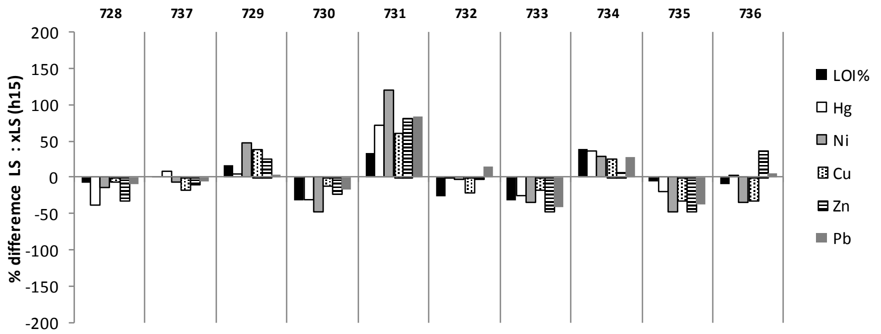

Online Resource 5: Percentage differences between littoral metal concentrations and mean (Hubers) of littoral sediments around calibration lakes.

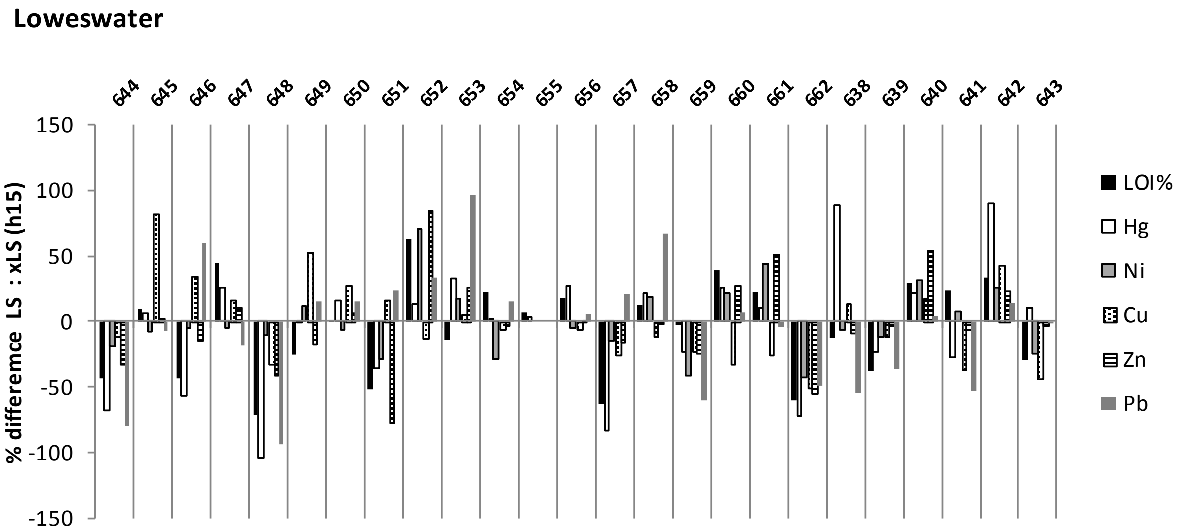

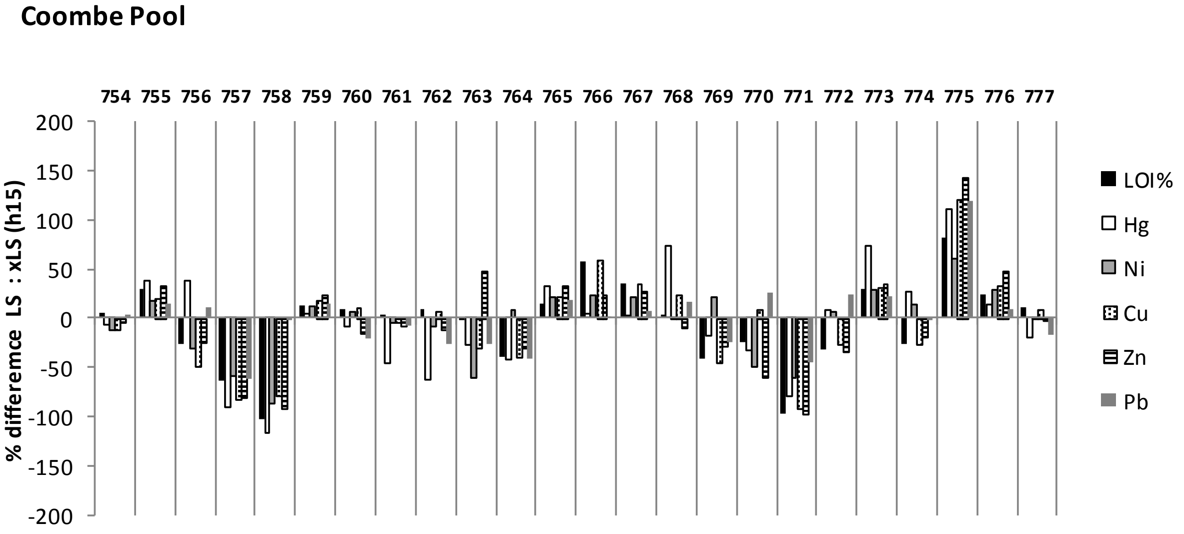

Supplement: Supplementary file 5 — (PDF 270 kb) [file 10661_2017_5946_MOESM5_ESM.pdf]
