# Supplementary material for: Using public participation to sample trace metals in lake surface sediments: the OPAL Metals Survey
Source: Environ Monit Assess. 2017 Apr 28;189(5):241. doi: 10.1007/s10661-017-5946-y (PMC5409918; doi:10.1007/s10661-017-5946-y)

Online Resource 6: (a) Organic content and metal concentrations (Hg ng g^{-1}, Ni, Cu, Zn, Pb µg g^{-1}) of sediment/soil compartments in the calibration lakes; means of littoral, deep water, soil and inflow stream sediments **(See Table 3).**

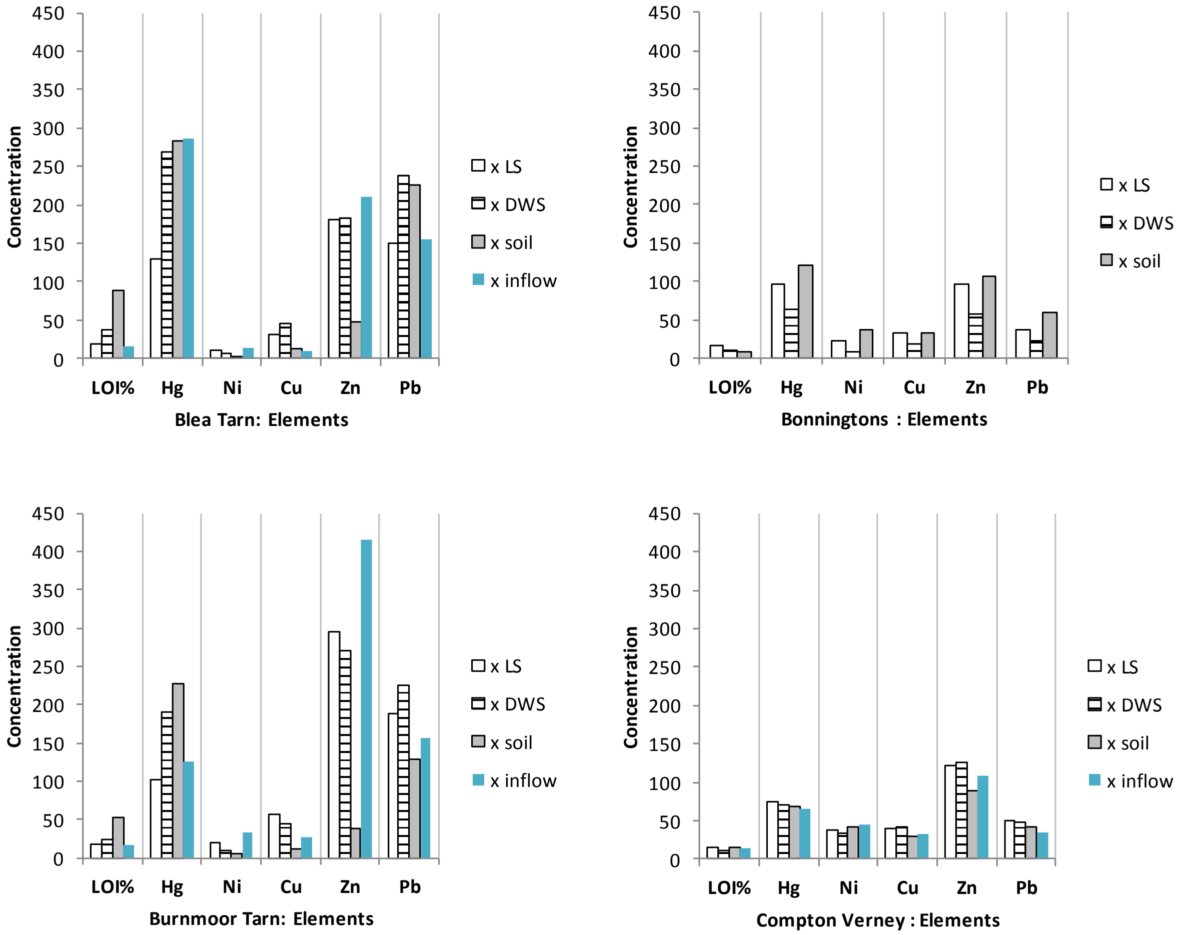

Online Resource 6: (b,c) Organic content and metal concentrations (Hg ng g^{-1}, Ni, Cu, Zn, Pb µg g^{-1}) of sediment/soil compartments in the calibration lakes; means of littoral, deep water, soil and inflow stream sediments **(See Table 3).**

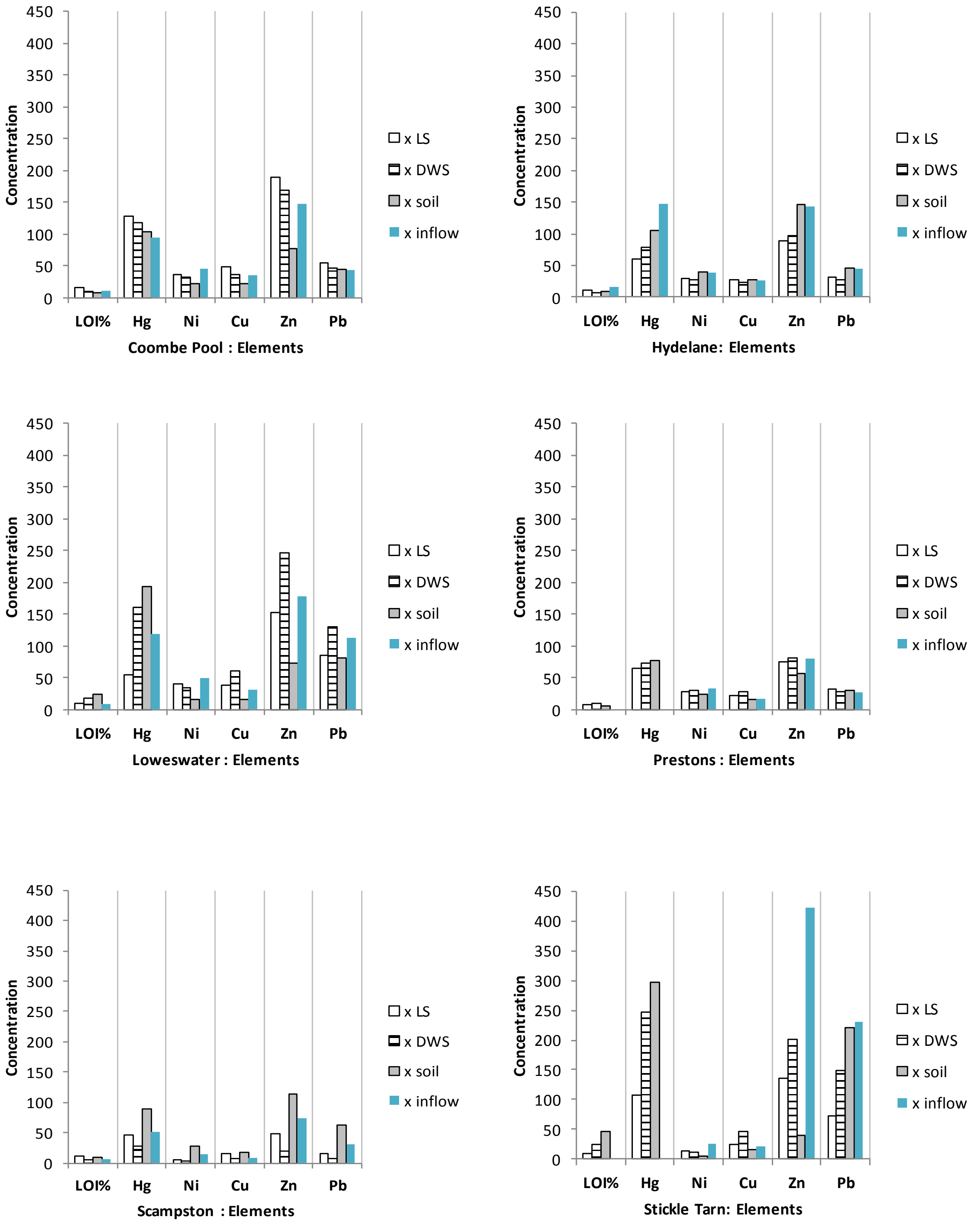

Supplement: Supplementary file 6 — (PDF 308 kb) [file 10661_2017_5946_MOESM6_ESM.pdf]
